# TMEM106B deficiency impairs cerebellar myelination and synaptic integrity with Purkinje cell loss

**DOI:** 10.1186/s40478-022-01334-7

**Published:** 2022-03-14

**Authors:** Tuancheng Feng, Lin Luan, Isabel Iscol Katz, Mohammed Ullah, Vivianna M. Van Deerlin, John Q. Trojanowski, Edward B. Lee, Fenghua Hu

**Affiliations:** 1 Department of Molecular Biology and Genetics, Weill Institute for Cell and Molecular Biology, Cornell University, 345 Weill Hall, Ithaca, NY 14853 USA; 2 Department of Pathology and Laboratory Medicine, Center for Neurodegenerative Disease Research, Institute On Aging, Perelman School of Medicine, University of Pennsylvania, Philadelphia, PA 19104 USA; 3 Translational Neuropathology Research Laboratory, Department of Pathology and Laboratory Medicine, Perelman School of Medicine, University of Pennsylvania, Philadelphia, PA 19104 USA

**Keywords:** TMEM106B, FTLD, Cerebellum, Lysosome, Myelination

## Abstract

TMEM106B, a type II lysosomal transmembrane protein, has recently been associated with brain aging, hypomyelinating leukodystrophy, frontotemporal lobar degeneration (FTLD) and several other brain disorders. TMEM106B is critical for proper lysosomal function and TMEM106B deficiency leads to myelination defects, FTLD related pathology, and motor coordination deficits in mice. However, the physiological and pathological functions of TMEM106B in the brain are still not well understood. In this study, we investigate the role of TMEM106B in the cerebellum, dysfunction of which has been associated with FTLD and other brain disorders. We found that TMEM106B is ubiquitously expressed in neurons in the cerebellum, with the highest levels in the Purkinje neurons. Aged TMEM106B-deficient mice show significant loss of Purkinje neurons specifically in the anterior lobe of the cerebellum. Increased microglia and astrocyte activation, as well as an accumulation of ubiquitinated proteins, p62 and TDP-43 were also detected in the cerebellum of aged TMEM106B deficient mice. In the young mice, myelination defects and a significant loss of synapses between Purkinje and deep cerebellar nuclei neurons were observed. Interestingly, TMEM106B deficiency causes distinct lysosomal phenotypes in different types of neurons and glia in the cerebellum and frontal cortex. In humans, TMEM106B rs1990622 risk allele (T/T) is associated with increased Purkinje neuron loss. Taken together, our studies support that TMEM106B regulates lysosomal function in a cell-type-specific manner and TMEM106B is critical for maintaining synaptic integrity and neural functions in the cerebellum.

**Supplementary Information:**

The online version contains supplementary material available at 10.1186/s40478-022-01334-7.

## Introduction

*TMEM106B*, a gene encoding a type II transmembrane protein of unknown function, was initially identified as a risk factor for frontotemporal lobar degeneration (FTLD) with *GRN* mutations [10, 15, 45]. Subsequently, *TMEM106B* polymorphisms have been associated with FTLD caused by *C9orf72* mutations [11, 17, 24, 44], cognitive impairment in Amyotrophic lateral sclerosis (ALS) [47] and Parkinson’s disease (PD) [42], and Alzheimer’s disease (AD) [34]. *TMEM106B* was also identified as a main risk factor for limbic-predominant age-related TDP-43 encephalopathy (LATE) [29] and *TMEM106B* is a primary determinant of brain aging [33]. More interestingly, a dominant D252N mutation in TMEM106B was recently identified as a cause of hypomyelinating leukodystrophy (HLD) [37, 49], a group of heritable neurodevelopmental disorders characterized by abnormal myelination in the central nervous system (CNS) [5]. In mouse models, TMEM106B deficiency was recently shown to cause myelination defects [14, 52], degeneration of Purkinje cells [32, 40], and FTLD related pathology in an age-dependent manner [13, 25]. Furthermore, TMEM106B deficiency exacerbates lysosome abnormalities and FTLD phenotypes in *Grn*^-/-^ mice [13, 48, 53].

At the cellular levels, TMEM106B is localized within the late endosome/lysosome [4, 6, 23] and regulates several aspects of lysosomal activities, including lysosomal morphology and function [4, 6, 23, 39], lysosome pH [6, 20, 22], lysosome exocytosis [22], lysosomal positioning within the cell [14], lysosomal trafficking in neuronal dendrites [36] and lysosomal trafficking across the axon initial segment (AIS) of axons [13, 25]. Thus, TMEM106B is critical for proper lysosomal function and closely linked to overall brain health.

TMEM106B-deficient mice exhibit motor coordination deficits [14, 25, 32, 40], which could be explained by lysosomal trafficking defects observed in motor neurons [13, 25], but also suggests a possible function of TMEM106B in the cerebellum. Recently, cerebellar dysfunction has been closely associated with FTLD and other neurodegenerative diseases [2, 3, 7, 8, 43]. However, the physiological and pathological functions of TMEM106B in the cerebellum are still unclear. Here we show that loss of TMEM106B leads to distinct lysosomal phenotypes in different cell types and results in myelination defects and the impairment of synaptic connectivity and axonal trafficking, and subsequent degeneration of Purkinje cells during aging.

## Material and methods

### Antibodies and reagents

The following antibodies were used in this study: mouse anti-Calbindin (Proteintech Group, 60004-1-Ig), rabbit anti-calbindin (Proteintech Group, 60004-1-Ig), mouse anti-parvalbumin (Sigma, SAB4200545), mouse anti-MAP2 (Proteintech Group, 67015-1-Ig), rabbit anti-MAP2 (Proteintech Group, 17490-1-AP), rabbit anti-CUX1 (Proteintech Group, 11733-1-AP), Goat anti-SYN (R&D Systems, AF5555), Chicken anti-NF-H (EnCor Biotechnology, CPCA-NF-H), mouse anti-NeuN (Millipore, MAB377), mouse anti-GAPDH (Proteintech Group, 60004-1-Ig), rabbit anti-p62 (MBL, PM045), mouse anti-Ubiquitin (BioLegend, 646302), rabbit anti-TDP-43 (Proteintech Group, 12892-1-AP), mouse anti-TDP43 (R&D Systems, MAB7778), rabbit anti-p-TDP-43 S403/S404 (COSMO BIO, CAC-TIP-PTD-P05), mouse anti-Ankyrin-G (Millipore, MABN466), rat anti-mouse LAMP1 (BD Biosciences, 553792), LAMP2 (Developmental Studies Hybridoma Bank, GL2A7-c), Goat anti-CathB (R&D Systems, AF965), Goat anti-CathD (R&D Systems, AF1029), Goat anti-CathL (R&D Systems, AF1515), mouse anti-GFAP (GA5) (Cell signaling, 3670S), rabbit anti IBA-1 (Wako, 01919741), and goat anti-AIF-1/Iba1 (Novus Biologicals, NB100-1028), mouse anti-PLP (Millipore, MAB388), mouse anti-MBP (Millipore, SMI-99), mouse anti-MOG (Abcam, ab203058), mouse anti-MAG (Proteintech Group, 14386-1-AP), and Goat anti-Olig2 (R&D Systems, AF2418). Rabbit anti-cathepsin D antibodies were a gift from Dr. William Brown at Cornell University. Rabbit anti-TMEM106B antibodies were characterized previously [4]. Alexa Fluor conjugated secondary antibodies (488/594/647 nm) were from Invitrogen. IRDye 680RD/800CW secondary antibodies were from Invitrogen and LI-COR Biosciences.

The following reagents were also used in the study: Odyssey blocking buffer (LI-COR Biosciences, 927-40000), protease inhibitors (Roche, 05056489001), TrueBlack^®^ Lipofuscin Autofluorescence Quencher (Biotium, #23007), Hoechst 33342 (Invitrogen), Pierce BCA Protein Assay Kit (Thermo scientific, 23225), Fluoromount-G (Thermo scientific, E113391), and O.C.T compound (Electron Microscopy Sciences, 62550-01).

### Mouse strains

TMEM106B knockout mice were produced using CRISPR/Cas9 genome editing with two guide RNAs targeting exon 3 of mouse *Tmem106b* gene, flanking the start codon  [14]. C57/BL6 mice were obtained from the Jackson Laboratory. Mixed male and female mice were used for this study. All animals (1–6 adult mice per cage) were housed in a 12 h light/dark cycle in the Weill Hall animal facility at Cornell University. All animal procedures have been approved by the Institutional Animal Care and Use Committee at Cornell University.

### Tissue preparation and Western blot analysis

Mice were perfused with PBS, and tissues were dissected and snap-frozen with liquid nitrogen and kept at − 80 °C. On the day of the experiment, frozen tissues were thawed and homogenized on ice with a bead homogenizer (Moni International) in a cold solution of RIPA buffer (150 mM NaCl, 50 mM Tris–HCl (pH 8.0), 1% Triton X-100, 0.5% sodium deoxycholate, 0.1% SDS) with proteinase and phosphatase inhibitors. After centrifugation at 14,000 × g for 15 min at 4 °C, supernatants were collected as the RIPA-soluble fraction. The insoluble pellets were washed with RIPA buffer and extracted in 2 × v/w of Urea buffer (7 M Urea, 2 M Thiourea, 4% CHAPS, 30 mM Tris, pH 8.5). After sonication, samples were centrifuged at 200,000 g at 24 °C for 1 h and the supernatant was collected as the Urea-soluble fraction. Protein concentrations were determined via BCA assay, then standardized. Equal amounts of protein were analyzed by western blotting using the indicated antibodies. Samples were run on 8%, 12%, or 15% polyacrylamide gels, then transferred to Immobilon-FL polyvinylidene fluoride membranes (Millipore Corporation). Membranes were blocked with either 5% non-fat milk in PBS or Odyssey Blocking Buffer (LI-COR Biosciences) for 1 h at room temperature and then incubated with primary antibodies, rocking overnight at 4 °C. Membranes were then washed with Tris-buffered saline with 0.1% Tween-20 (TBST) for 3 times, 5 min each, and incubated with fluorescently tagged secondary antibodies (LI-COR Biosciences) for 1 h at room temperature, and then followed by three washes, 10 min each. Membranes were scanned using an Odyssey Infrared Imaging System (LI-COR Biosciences). Densitometry was performed using Image Studio (LI-COR Biosciences) and Image J.

### Immunofluorescence staining, image acquisition, and analysis

Mice were perfused with cold PBS, and tissues were post-fixed with 4% paraformaldehyde. After dehydration, the brain tissues were embedded in the O.C.T compound (Electron Microscopy Sciences). 15-µm-thick brain sections were cut with cryotome. Tissue sections were permeabilized and blocked with 0.1% saponin in Odyssey blocking buffer or 0.2% Triton X-100 in 1 × PBS with 5% horse serum at room temperature for 1 h. Sections were then incubated with primary antibodies overnight at 4 °C. The next day, sections were then incubated with secondary fluorescence antibodies and Hoechst at room temperature for 1 h. After washing, the sections were mounted using a mounting medium (Vector laboratories). To block the autofluorescence, all sections were incubated with 1 × TrueBlack Lipofuscin Autofluorescence Quencher (Biotium) in 70% ethanol for 30 s at room temperature before or after the staining process. Antigen retrieval was performed by microwaving in sodium citrate buffer (pH 6.0) for 10 min. Images were acquired on a CSU-X spinning disc confocal microscope (Intelligent Imaging Innovations) with an HQ2 CCD camera (Photometrics) using 40 × and 100 × objectives. Lower magnification images were captured by 10 × or 20 × objectives on a Leica DMi8 microscope. The quantitative analysis of fluorescence images was performed using ImageJ. Five to ten different random images were captured, and the fluorescence intensity was measured directly with ImageJ after a threshold application. Data from ≥ 3 brains in each genotype were used for quantitative analysis.

### Hematoxylin and eosin (H&E) staining and Purkinje cell number analysis in human tissues

Human brain tissues were obtained from the Center for Neurodegenerative Disease Brain Bank at the University of Pennsylvania with authorization for autopsy provided by patients’ next-of-kin. Neuropathological diagnoses were made in accordance with consensus diagnostic criteria. Cerebellar tissues were identified that lacked neurodegenerative disease neuropathologic inclusions (i.e. absence of β-amyloid, tau, α-synuclein, or TDP-43 aggregates). Detailed information is provided in Additional file 1: Table 1.

Human cerebellum sections were paraffin-embedded for H&E staining. Paraffin-embedded tissues sections (6 μm) were stained with hematoxylin and eosin as described [41]. Whole slide images were obtained using a digital slide scanner (Aperio AT2, Leica Biosystem, Wetzlar, Germany) at 20 × magnification. To count the Purkinje cell number, lines were drawn along the Purkinje cell layer to measure the distance (mm), and the number of Purkinje cells along the line was counted by a researcher blinded by the genotypes. The density of the Purkinje cell was calculated as the total cell number/distance.

### Statistical analysis

All statistical analyses were performed using GraphPad Prism 8. All data are presented as mean ± SEM. Statistical significance was assessed by paired or unpaired t test (for two group comparisons). P values less than or equal to 0.05 were considered statistically significant. *p < 0.05; **p < 0.01; ***p < 0.001; ****p < 0.0001.

## Results

### Purkinje cell degeneration, glial activation, and autophagy defects in the cerebellum of aged TMEM106B-deficient mice

TMEM106B-deficient mice exhibit motor coordination deficits [14, 25, 32, 40], suggesting a possible function of TMEM106B in the cerebellum. To determine the expression pattern of TMEM106B in the cerebellum, we stained cerebellum sections from adult mice with antibodies against TMEM106B and the lysosomal enzyme, cathepsin D. Sections from age-matched *Tmem106b*^*−/−*^ mice were used as controls to confirm the specificity of TMEM106B antibodies. We found that TMEM106B is expressed in many different types of neurons in the cerebellum and co-localizes with the lysosomal marker cathepsin D, with its highest expression levels observed in Purkinje cells (Additional file 1: Fig. 1).

**Fig. 1:**
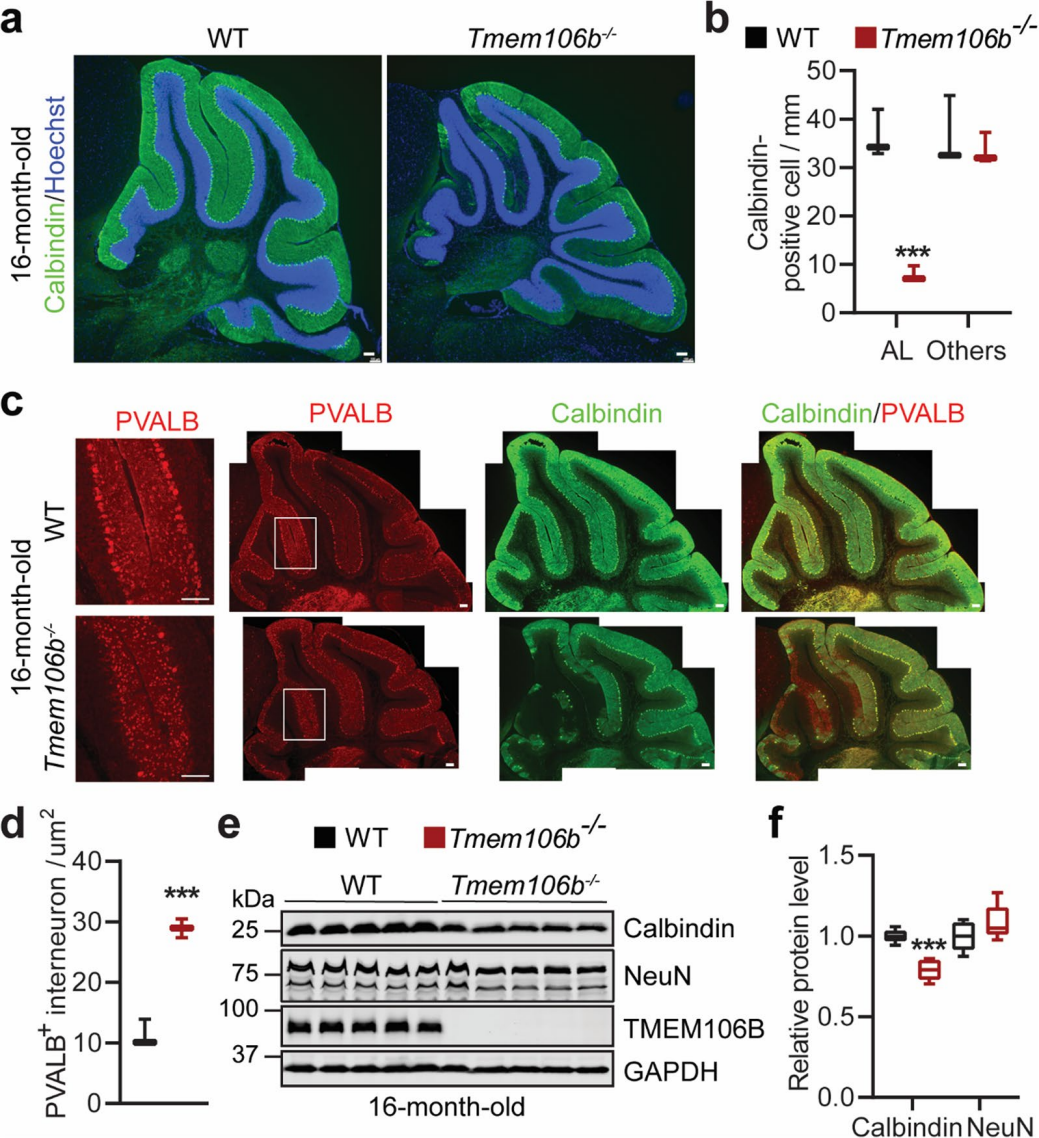
Degeneration of Purkinje cells in aged *Tmem106b*^*−/−*^ mice. **a**, **b** Cerebellar sections from 16‐month‐old WT and *Tmem106b*^*−/−*^ mice were stained with anti-calbindin antibody and Hoechst. Purkinje cell numbers in the anterior lobe (AL) and other regions (Others) were quantified in **b**. Scale bar = 100 µm. n = 3–4, ***, *p* < 0.001, unpaired t-test. **c**, **d** Cerebellar sections from 16‐month‐old WT and *Tmem106b*^*−/−*^ mice were immuno-stained with antibodies of calbindin and parvalbumin (PVALB). Zoom-in images show PVALB-positive interneurons in the MCL adjacent to the region with Purkinje cell loss. The number of PVALB-positive interneurons in the molecular layer of the anterior lobe (AL) was quantified in **d**. Scale bar = 100 µm. n = 3, ***, *p* < 0.001, unpaired t-test. **e**,** f** Western blot analysis of the protein level of Calbindin, NeuN, and TMEM106B in 16-month-old WT and *Tmem106b*^*−/−*^ cerebellar lysates. GAPDH was used as an internal control. Relative protein levels were quantified in **f**. n = 5, ***, *p* < 0.001, unpaired t-test

Consistent with previous reports [32, 40], we observed a significant loss of Purkinje cells specifically in the anterior lobe (AL) of the cerebellum in the 16-month-old *Tmem106b*^*−/−*^ mice (Fig. 1a, b). Surprisingly, the density of parvalbumin (PVALB)-positive interneurons is dramatically increased in the molecular layer (ML) adjacent to the region with Purkinje cell loss (Fig. 1c, d). Moreover, western blot analysis showed a decrease in the levels of Calbindin, which is commonly used as a marker for Purkinje cells, in cerebellar lysates from the 16-month-old *Tmem106b*^*−/−*^ mice, with no changes in the levels of NeuN, a general marker for neurons (Fig. 1e, f). This phenotype can be observed at 10-month-old *Tmem106b*^*−/−*^ mice, but not at 2-or 5-month-old mice (Additional file 1: Fig. 2a–e). Together, these data suggest that TMEM106B deficiency results in an opposing change in the density of Purkinje cells and PVALB-positive interneurons in an age-dependent manner.

Accompanied with Purkinje neuron degeneration, a drastic increase in the intensity of microglial marker IBA-1 and the astrocyte marker GFAP was observed in the cerebellum of 16-month-old *Tmem106b*^*−/−*^ mice (Additional file 1: Fig. 3a and b). This increase in GFAP levels can also be detected in cerebellar lysates using western blot analysis (Additional file 1: Fig. 3c and d), but the phenotype was not observed in the cerebellum of 2-month-old *Tmem106b*^*−/−*^ mice (Additional file 1: Fig. 3e and f). Thus, TMEM106B deletion leads to an age-dependent glia activation in the cerebellum.

**Fig. 2:**
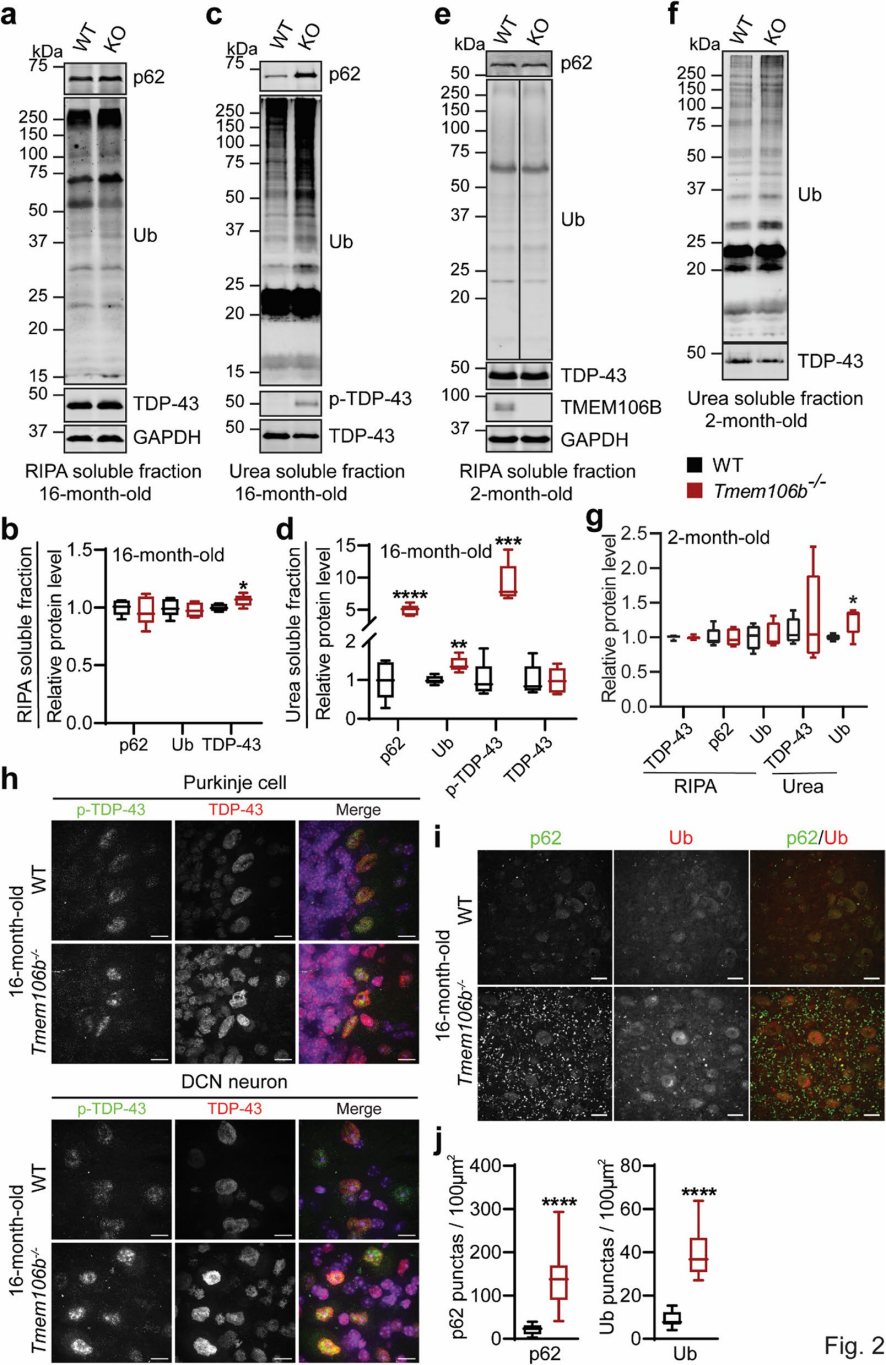
ALS/FTLD related pathological changes in the cerebellum of aged *Tmem106b*^*−/−*^ mice. **a**–**d** Western blot analysis of p62, ubiquitin (Ub), TDP‐43, and p‐TDP-43 in RIPA—(**a**, **b**) and urea—(**c**, **d**) soluble fractions from the cerebellum of 16‐month‐old WT and *Tmem106b*^*−/−*^ mice. Protein levels were quantified and normalized to GAPDH. n = 5, *, *p* < 0.05; **, *p* < 0.01, ***, *p* < 0.001, ****, *p* < 0.0001, unpaired t-test. **e**–**g** Western blot analysis of p62, ubiquitin (Ub), and TDP‐43 in RIPA—(**e**, **g**) and urea—(**f**, **g**) soluble fractions from the cerebellum of 2-month‐old WT and *Tmem106b*^*−/−*^ mice. Protein levels were quantified and normalized to GAPDH. n = 5, *, *p* < 0.05, unpaired t-test. **h** Immunostaining of TDP‐43 and p‐TDP-43 in the cerebellar sections from 16‐month‐old WT and *Tmem106b*^*−/−*^ mice. Representative images from the Purkinje cell layer and deep cerebellar nuclei (DCN) are shown. **i**, **j** Immunostaining of p62 and ubiquitin (Ub) in the cerebellar sections from 16‐month‐old WT and *Tmem106b*^*−/−*^ mice. The number of p62 or Ub positive puncta was quantified in (**j**). n = 4. ****, *p* < 0.0001, unpaired t-test

Previously, we and others have shown that loss of TMEM106B results in age-dependent-ALS/FTLD related pathological changes, including an increase in the levels of ubiquitinated proteins, autophagy adaptor protein p62, and phosphorylated TDP-43 in both the brain and the spinal cord [13, 25]. TMEM106B has also been shown to modify TDP-43 pathology in human ALS brain and cell-based models of TDP-43 proteinopathy [26]. We also observe ubiquitin, p62 and TDP-43 pathology in the cerebellum of TMEM106B deficient mice (Fig. 2). A significant accumulation of ubiquitinated proteins, the autophagy adaptor protein p62, and phosphorylated TDP-43 (S403/S404) in the RIPA-insoluble fraction were detected in the cerebellar lysates of 16-month-old *Tmem106b*^*−/−*^ mice (Fig. 2a–d). The levels of TDP-43 were also slightly increased in RIPA-soluble fractions (Fig. 2a, b). The accumulation of ubiquitinated proteins was also observed in RIPA-insoluble fractions in the cerebellar lysates from 2-month-old *Tmem106b*^*−/−*^ mice (Fig. 2e–g), indicating an early defect in autophagy. To determine which cell type in the cerebellum exhibits this phenotype, we stained cerebellar sections from 16-month-old *Tmem106b*^*−/−*^ mice with antibodies against TDP-43, phosphorylated TDP-43 (S403/S404), p62, and ubiquitin (Ub). An increase in phosphorylated TDP-43, as well as total TDP-43 signals, was observed in Purkinje cells and deep cerebellar nucleus (DCN) neurons (Fig. 2h), while the accumulation of p62- and ubiquitin-positive aggregates is most obvious in the DCN region of aged *Tmem106b*^*−/−*^ mice (Fig. 2i, j). In addition, p62 positive puncta overlap with calbindin-positive Purkinje cell axons in the DCN (Additional file 1: Fig. 4), suggesting that these p62 signals might be derived from axon terminals of dying Purkinje cells. Thus, ablation of TMEM106B causes defects in protein homeostasis in the cerebellum, resulting in the accumulation of ubiquitinated proteins and autophagy adaptor p62.

### Myelination defects and axonal degeneration of Purkinje cells in young TMEM106B-deficient mice

**Fig. 3:**
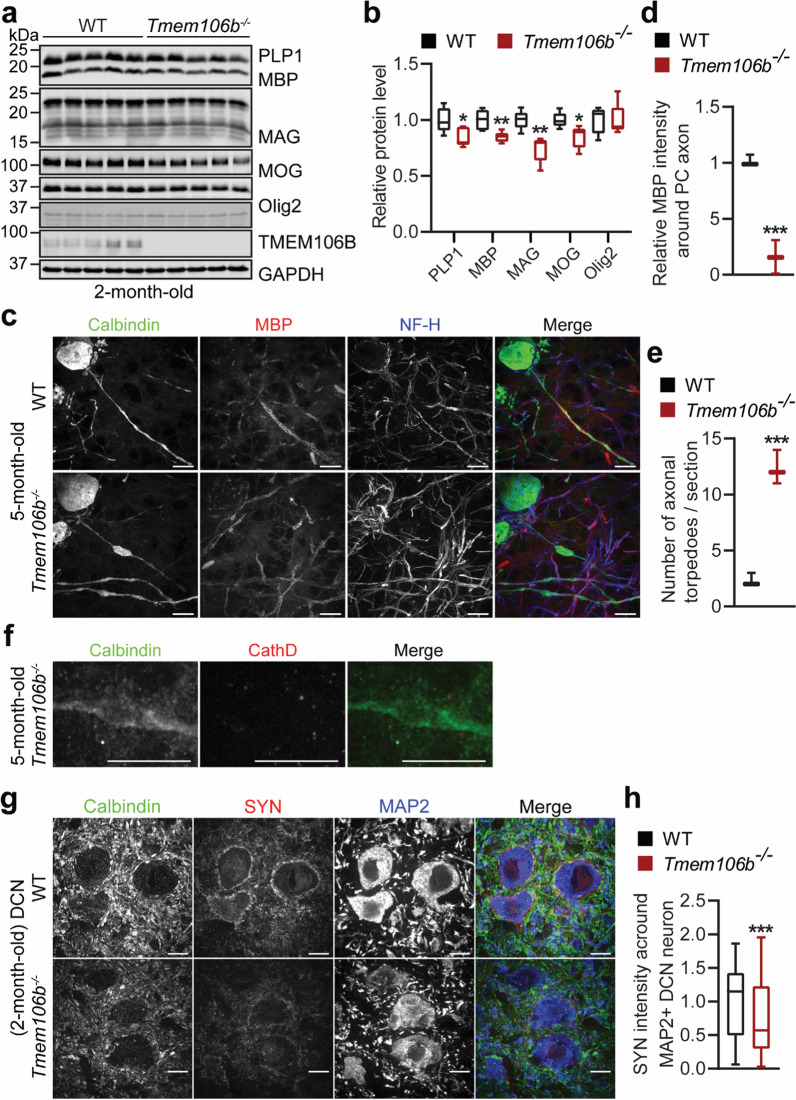
Myelination defects, axonal degeneration of Purkinje cells and disruption of cerebellar cortico-nuclear connection in young *Tmem106b*^*−/−*^ mice. **a**, **b** Western blot analysis of myelin proteins and GAPDH in 2-month-old WT and *Tmem106b*^*−/−*^ cerebellar lysates. Protein levels were quantified and normalized to GAPDH in **b**. n = 5, *, *p* < 0.05, **, *p* < 0.01, unpaired t-test. **c**–**e** Cerebellar sections from 5‐month‐old WT and *Tmem106b*^*−/−*^ mice were co-stained with anti-calbindin, myelin basic protein (MBP), and NF-H antibodies. MBP intensity around Purkinje cell axon and the number of giant torpedos in the axon of Purkinje cells were quantified in **d** and **e**, respectively. Scale bar = 10 µm. n = 3–4, ***, *p* < 0.001, unpaired t-test. **f** Cerebellar sections from 5‐month‐old *Tmem106b*^*−/−*^ mice were co-stained with anti-calbindin and Cath D antibodies. Scale bar = 10 µm. **g**, **h** Cerebellar sections from 2‐month‐old WT and *Tmem106b*^*−/−*^ mice were immunostained with antibodies of calbindin, synaptophysin (SYN, presynaptic marker), and MAP2. The intensity of SYN around MAP2-positive soma in the DCN region was quantified in **h**. Scale bar = 10 µm. n = 3, ***, *p* < 0.001, non-parametric test (Mann Whitney test)

Since TMEM106B mutation is associated with hypomyelinating leukodystrophy (HLD) [37, 49] and TMEM106B deficiency in mice causes myelination defects [14, 52], we examined myelination defects in the cerebellum of *Tmem106b*^*−/−*^ mice. The levels of several myelin proteins, including proteolipid protein 1 (PLP1), myelin basic protein (MBP), myelin oligodendrocyte glycoprotein (MOG), and myelin-associated glycoprotein (MAG) were significantly decreased in the cerebellar lysates from 2-month—(Fig. 3a, b) and 5–6-month-old (Additional file 1: Fig. 5a and b) *Tmem106b*^*−/−*^ mice, whereas the levels of Olig2, a transcription factor expressed in both immature and mature oligodendrocytes, were not affected by TMEM106B ablation (Fig. 3a, b, and Additional file 1: Fig. 5a and b). Unsurprisingly, decreased levels of myelin proteins were also detected in the 16-month-old *Tmem106b*^*−/−*^ mouse cerebellum (Additional file 1: Fig. 5c and d). To examine myelination defects in more detail, we performed immunostaining with calbindin, MBP, and neurofilament (NF-H) antibodies to examine myelination defects of Purkinje axons. The intensity of MBP surrounding the axon of Purkinje cells was significantly reduced in *Tmem106b*^*−/−*^ mice (Fig. 3c, d). In addition, the de-myelinated axons show a giant cathepsin D-negative torpedo in 5-month-old *Tmem106b*^*−/−*^ mice (Fig. 3c, e, f), indicating axon swelling. This phenotype is enhanced in 16-month-old *Tmem106b*^*−/−*^ mice (Additional file 1: Fig. 6a and b) but is not observed in 2-month-old *Tmem106b*^*−/−*^ mice (Additional file 1: Fig. 6c), indicating that this is an age-related phenotype. These findings support that TMEM106B deficiency leads to myelination defects, swelling, and degeneration of Purkinje axons, which might contribute to Purkinje cell death in *Tmem106b*^*−/−*^ mice during aging. While we have only examined the myelination defects of Purkinje axons in detail, it is highly likely that other myelinated axons are affected by the loss of TMEM106B as well, given the overall decrease in the levels of myelinated proteins in the *Tmem106b*^*−/−*^ mouse cerebellum.

### Disruption of cerebellar cortico-nuclear connection in young Tmem106b^−/−^ mice

Purkinje cells constitute the sole output of the cerebellar cortex, whereas cerebellar nuclei constitute the sole output of the entire cerebellum [9]. Purkinje cells receive excitatory input from the collaterals of the mossy and climbing fibers, make inhibitory connections onto the cerebellar nuclei, and inhibit the activity of the deep cerebellar nuclei [27]. To examine the connection between Purkinje cells and deep cerebellar nuclei, we performed immunostaining with antibodies against calbindin, synaptophysin (SYN, presynaptic marker), and MAP2 to analyze the synaptic formation between Purkinje cell axon and the soma of DCN neurons. A significant reduction of SYN levels around MAP2-positive soma of DCN neurons was observed in the cerebellum of *Tmem106b*^*−/−*^ mice at 2-month-old (Fig. 3g, h), suggesting that TMEM106B ablation leads to an early defect in the synaptic connection between Purkinje axons and deep cerebellar nuclei neurons, resulting in the disruption of cerebellar cortico-nuclear connections in *Tmem106b*^*−/−*^ cerebellum. The disruption of synaptic connection might also contribute to axonal swelling and degeneration of Purkinje cells.

### Distinct lysosomal phenotypes in different cell types in the cerebellum of TMEM106B-deficient mice

**Fig. 4:**
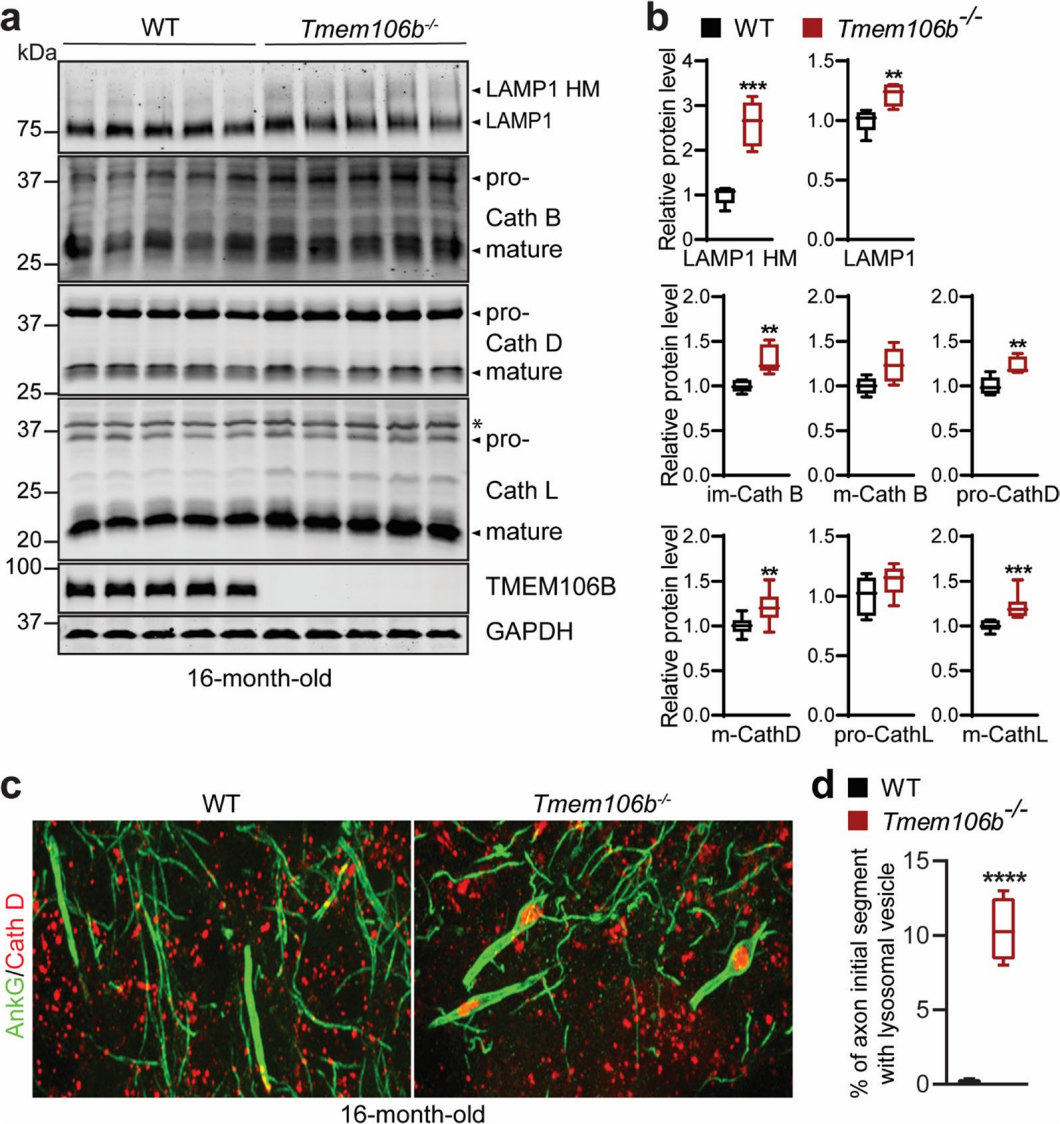
Lysosome and lysosomal trafficking defects in the cerebellum of aged *Tmem106b*^*−/−*^ mice. **a**, **b** Western blot analysis of lysosomal proteins and GAPDH in 16-month-old WT and *Tmem106b*^*−/−*^ cerebellar lysates. Protein levels were quantified and normalized to GAPDH in **b**. HM: high molecular weight. n = 5, **, *p* < 0.01, ***, *p* < 0.001, unpaired t-test. Asterisk indicates non‐specific bands. **c**, **d** Immunostaining of AnkG and Cath D in the cerebellar sections from 16‐month‐old WT and *Tmem106b*^*−/−*^ mice. Representative images from the Purkinje cell layer are shown. The percentage of axon initial segments (AISs) with Cath D-positive lysosomal vesicles were quantified in **d**. n = 3. ****, *p* < 0.0001, unpaired t-test

Since TMEM106B is a lysosomal protein, we next examined the lysosomal phenotypes in the cerebellum of TMEM106B deficient mice. A slight increase in the protein levels of lysosomal cathepsins (D and L), but not lysosomal membrane protein LAMP1 and LAMP2, was observed in the cerebellar lysates from 6-month-old *Tmem106b*^*−/−*^ mice (Additional file 1: Fig. 7). A significant increase in the protein levels of both LAMP1 and cathepsins (B, D, and L) was detected in the cerebellar lysates from 16-month-old *Tmem106b*^*−/−*^ mice (Fig. 4a, b). A dramatic increase of high molecular weight form of LAMP1was also observed in the 16-month-old cerebellar lysates, which might be due to the change in the glycosylation pattern of LAMP1 (Fig. 4a, b). Next, we examined lysosomal phenotypes in individual cell types more carefully using immuno-staining. TMEM106B deficiency results in the accumulation of lysosomal vacuoles at the distal end of the axon initial segment in motor neurons [13, 25]. This phenotype is also observed in the Purkinje cells in 16-month-old *Tmem106b*^*−/−*^ mice (Fig. 4c, d), consistent with previous reports from other groups [32, 40]. However, this phenotype was not observed in young mice (data not shown). This suggests a lysosomal trafficking defect in Purkinje cell axons in aged *Tmem106b*^*−/−*^ mice.

**Fig. 5:**
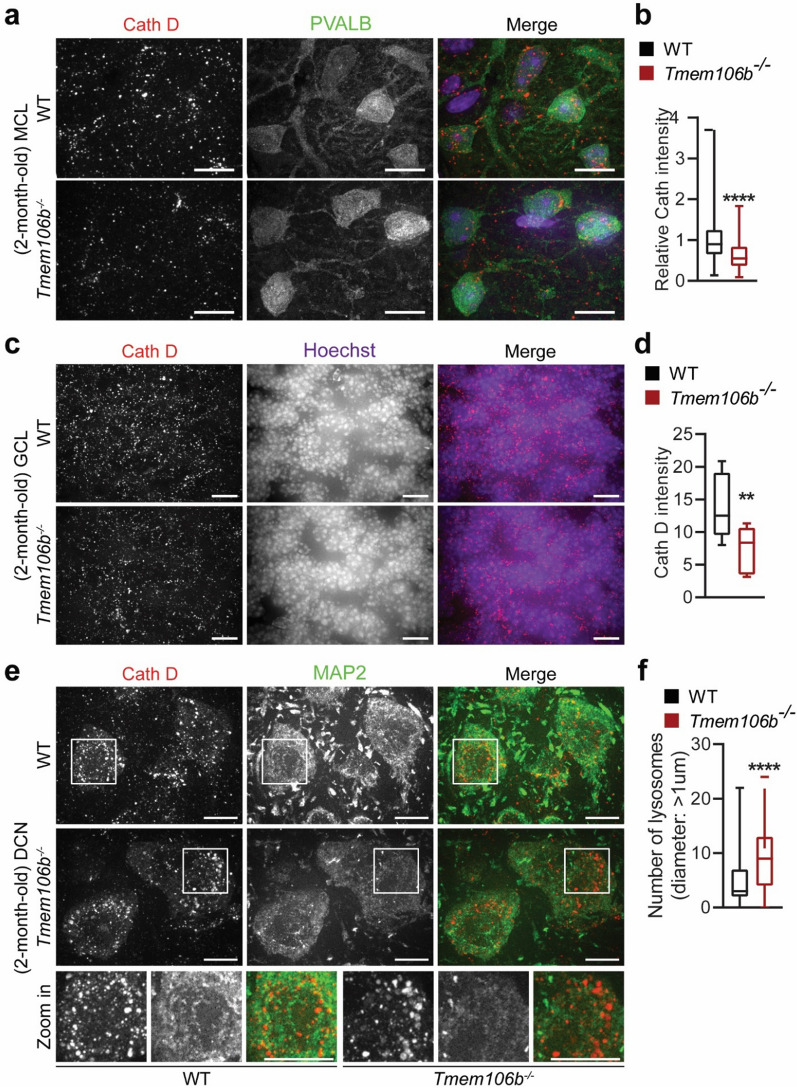
Distinct lysosomal phenotypes among different types of neurons in the cerebellum of young *Tmem106b*^*−/−*^ mice. **a**, **b** Immunostaining of PVALB and Cathepsin D (Cath D) in cerebellar sections from 2-month‐old WT and *Tmem106b*^*−/−*^ mice. Representative images from the molecular cell layer are shown. The intensity of Cath D in PVALB-positive interneurons was quantified in **b**. n = 3. ****, *p* < 0.0001, unpaired t-test. Scale bar = 10 μm. **c**, **d** Immunostaining of Cathepsin D (Cath D) and Hoechst in cerebellar sections from 2-month‐old WT and *Tmem106b*^*−/−*^ mice, and images were captured from granule cell layer. The intensity of Cath D in the granule cell layer was quantified in **d**. n = 3. **, *p* < 0.001, unpaired t-test. Scale bar = 10 μm. **e**, **f** Immunostaining of Cathepsin D (Cath D) and MAP2 in cerebellar sections from 2-month‐old WT and *Tmem106b*^*−/−*^ mice. Representative images from the DCN region are shown. The intensity of Cath D in MAP2-positive DCN neurons was quantified in **f**. Zoom-in image shows the enlarged lysosomes in 2-month‐old *Tmem106b*^*−/−*^ mice compared with WT mice. n = 3. ****, *p* < 0.0001, unpaired t-test. Scale bar = 10 μm

**Fig. 6:**
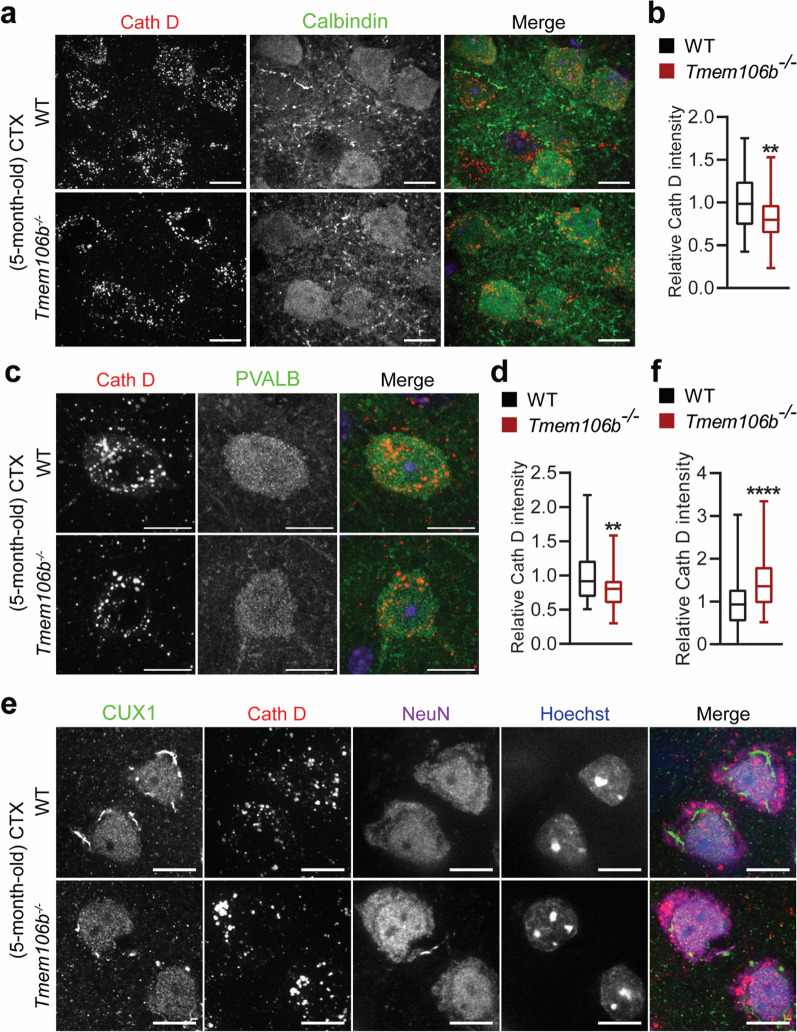
Distinct lysosomal phenotypes among different types of neurons in the cortex (CTX) in young *Tmem106b*^*−/−*^ mice. **a**, **b** Immunostaining of Calbindin and Cath D in brain sections from 5-month‐old WT and *Tmem106b*^*−/−*^ mice, and images were captured from the frontal cortex. The intensity of Cath D in Calbindin-positive neurons was quantified in **b**. n = 3. **, *p* < 0.01, unpaired t-test. Scale bar = 10 μm. **c**, **d** Immunostaining of PVALB and Cathepsin D (Cath D) in brain sections from 5-month‐old WT and *Tmem106b*^*−/−*^ mice. Representative images from the frontal cortex are shown. The intensity of Cath D in PVALB-positive interneurons was quantified in **d**. n = 3. **, *p* < 0.01, unpaired t-test. Scale bar = 10 μm. **e**, **f** Immunostaining of CUX1, Cath D, and NeuN in brain sections from 5-month‐old WT and *Tmem106b*^*−/−*^ mice. Representative images from the frontal cortex are shown. The intensity of Cath D in CUX1-positive excitatory neuron was quantified in **f**. n = 3. ****, *p* < 0.0001, unpaired t-test. Scale bar = 10 μm

To determine the early lysosomal changes in different neuronal types in *Tmem106b*^*−/−*^ mice, we performed co-staining of cathepsin D (CathD) and PVALB or MAP2 in the cerebellar sections from 2-month-old wild type (WT) and *Tmem106b*^*−/−*^ mice. Interestingly, the intensity of Cath D was significantly reduced in PVALB-positive interneurons in the molecular layer (Fig. 5a, b) and granule cells (Fig. 5c, d). In contrast, MAP2-positive deep cerebellar nuclei neurons exhibited enlarged lysosomes and no significant changes in Cath D levels (Fig. 5e, f), indicating that the loss of TMEM106B results in distinct lysosomal phenotypes among different types of neurons in the cerebellum, with reduced Cath D levels in the PVALB positive inhibitory interneurons and cerebellar granule neurons and enlarged lysosomes in the excitatory DCN neurons. This differential lysosomal phenotype was also observed in the cortex, with Cath D levels significantly decreased in calbindin- or PVALB-positive inhibitory neurons (Fig. 6a–d), and increased in CUX1-positive excitatory neurons (Fig. 6e, f) in the 5-month-old *Tmem106b*^*−/−*^ mice. Lysosome clustering and enlargement were also observed in the CUX1-positive excitatory neurons but not PVALB-positive interneurons in the cortex (Fig. 6e, f). These data support that TMEM106B deficiency has different effects on different types of neurons in the cerebellum and cortex.

**Fig. 7:**
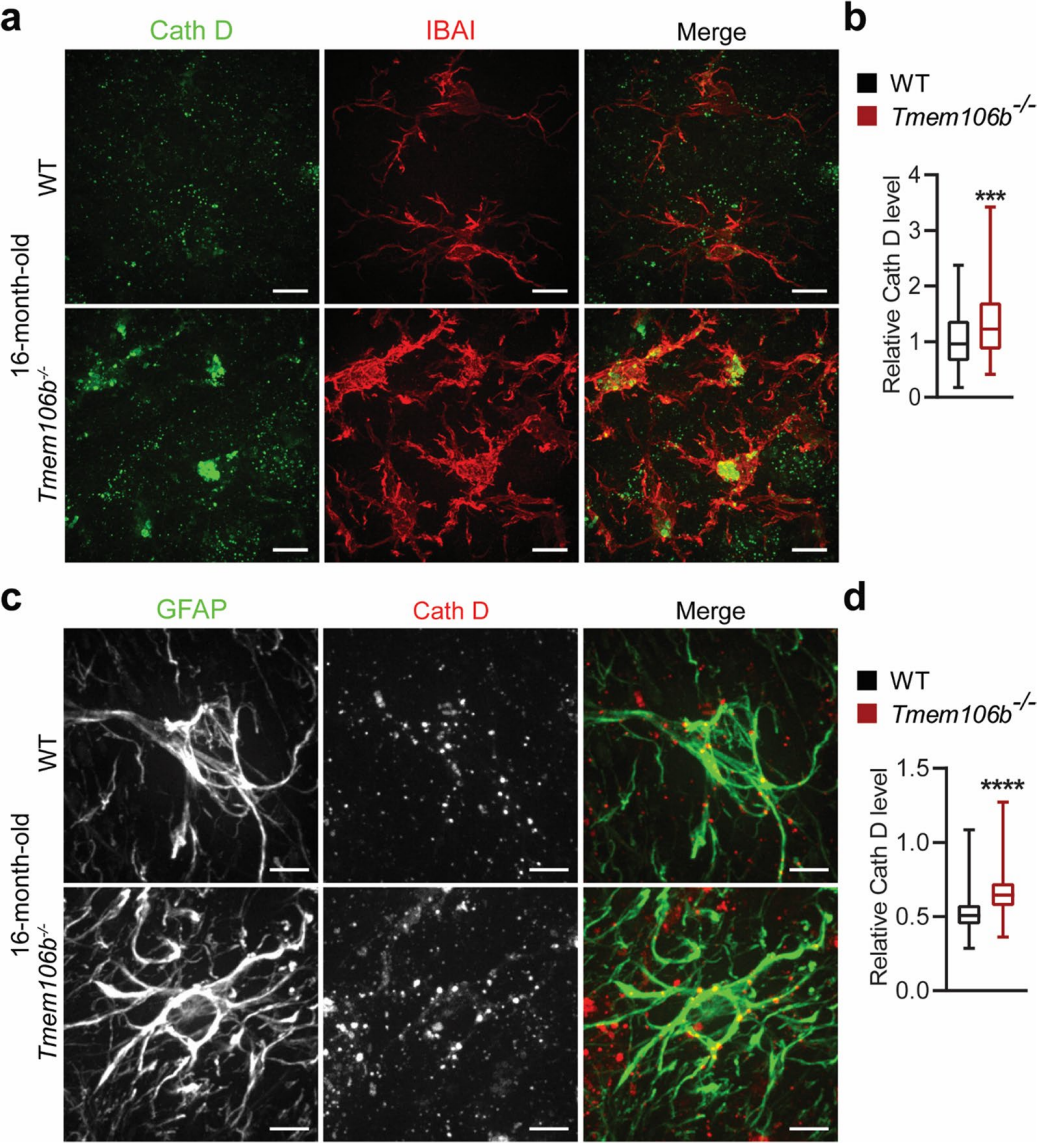
Lysosome defects in glial cells in the cerebellum of aged *Tmem106b*^*−/−*^ mice. **a**, **b** Cerebellar sections from 16-month‐old WT and *Tmem106b*^*−/−*^ mice were immunostained with IBA1 and Cath D antibodies. Representative images from the DCN region are shown. The intensity of Cath D in IBA1-positive microglia was quantified in **b**. n = 3. ***, *p* < 0.001, unpaired t-test. Scale bar = 10 μm. **c**, **d** Cerebellar sections from 16-month‐old WT and *Tmem106b*^*−/−*^ mice were immunostained with GFAP and Cath D antibodies. Representative images from the DCN region are shown. The intensity of Cath D in GFAP-positive astrocytes was quantified in **d**. n = 3. ****, *p* < 0.0001, unpaired t-test. Scale bar = 10 μm

To determine lysosomal phenotypes in microglia and astrocyte of *Tmem106b*^*−/−*^ mice, we performed co-staining of CathD and microglial marker IBA1 or astrocyte marker GFAP. Interestingly, CathD protein levels were significantly increased in IBA1-positive microglia in the DCN region of 16-month-old *Tmem106b*^*−/−*^ mice compared to WT controls (Fig. 7a, b). In addition, lysosomes were clustered and enlarged in *Tmem106b*^*−/−*^ microglia (Fig. 7a, b). A mild increase of CathD protein levels was also observed in GFAP positive astrocytes in the DCN region of 16-month-old *Tmem106b*^*−/−*^ mice (Fig. 7c, d), but the lysosomal distribution appears normal compared to WT controls. However, no obvious lysosomal phenotypes were observed in microglia and astrocyte in the young *Tmem106b*^*−/−*^ mice (data not shown). This suggests that the microglial phenotype in the aged *Tmem106b*^*−/−*^ mice could be due to increased phagocytosis of cell debris during aging and Purkinje cell death. Nevertheless, our data support that TMEM106B ablation leads to distinct lysosomal phenotypes in different cell types.

### *TMEM106B* rs1990622 risk allele is associated with increased Purkinje cell degeneration in humans

**Fig. 8:**
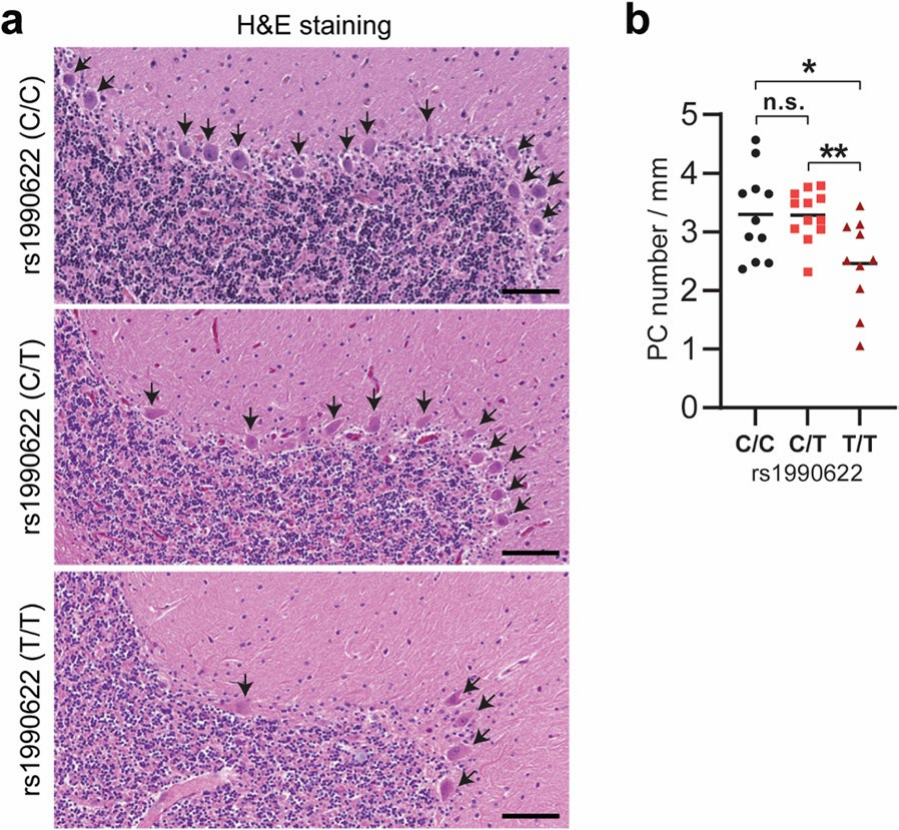
*TMEM106B* rs1990622 risk allele is associated with Purkinje cell loss in humans. **a** H&E staining was performed in human cerebellum sections from cohorts with different TMEM106B rs1990622 genotypes (C/C, C/T, and T/T). Representative images from each genotype are shown. **b** Number of Purkinje cells was counted in the cerebellar section, and the density of Purkinje cells along the Purkinje cell layer was calculated. n = 10–13 for each genotype, n.s., not significant, *, *p* < 0.05, **, *p* < 0.001, unpaired t-test. Scale bar = 100 µm

*TMEM106B* was initially discovered as a main risk factor for FTLD with TDP-43 pathology, especially in *GRN* mutant carriers [10, 15, 45]. The rs1990622 single nucleotide polymorphism (SNP), in the 3’ untranslated region (UTR), is most commonly associated with modulating the disease risk; almost all other disease-associated *TMEM106B* SNPs are in linkage disequilibrium with rs1990622, making it a sentinel SNP [10, 15, 16, 24, 31, 45, 46]. The major allele is also found to be associated with faster cognitive decline in Parkinson’s disease (PD) [42], more advanced TDP-43 pathology in AD [34] and old persons without FTLD [50], and an increased risk for hippocampal sclerosis in aging (HS-aging) [30] and LATE [19, 29]. To determine whether TMEM106B polymorphisms are associated with the loss of Purkinje cells during aging in humans, we performed H&E staining of postmortem human cerebellum sections from donors without evidence of cerebellar neurodegenerative disease proteinopathy (Fig. 8a, Additional file 1: Table 1). The number of Purkinje cells was quantified in brains with different TMEM106B rs1990622 genotypes. Interestingly, we found that homozygotes of the rs1990622 risk T allele (T/T) have a significantly lower number of Purkinje cells compared to homozygotes of the rs1990622 protective C allele (C/C) and heterozygotes (Fig. 8a. b). Thus, the TMEM106B risk allele is associated with increased Purkinje cell degeneration in humans.

## Discussion

**Fig. 9:**
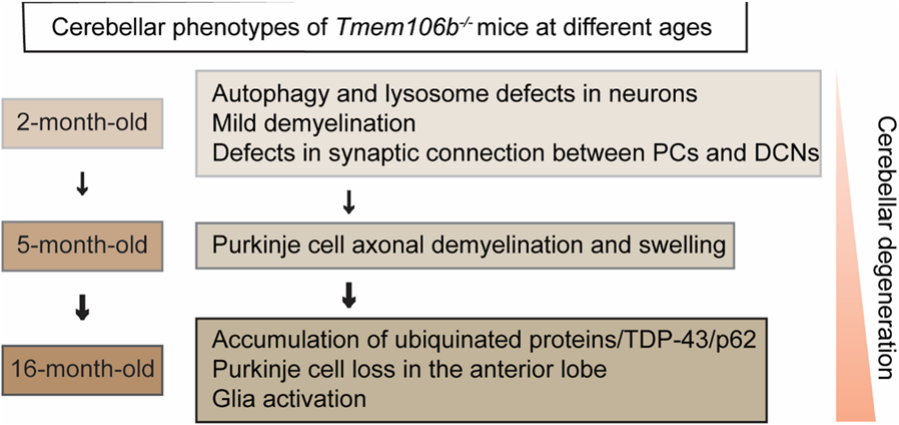
A diagram summarizing cerebellar pathologies in *Tmem106b*^*−/−*^ mice with age. At 2 months of age, *Tmem106b*^*−/−*^ young mice exhibit lysosome and myelination defects, and a significant loss of synapses between Purkinje and deep cerebellar nuclei neurons. Purkinje cell axonal demyelination and swelling are observed at 5 months of age. Significant loss of Purkinje neurons specifically in the anterior lobe of the cerebellum are observed in 16-month of age, accompanied by increased microglia and astrocyte activation, and the accumulation of ubiquitinated proteins, p62, and TDP-43

TMEM106B is intimately linked with brain aging and brain disorders [12]. Our analysis of cerebellar phenotypes in TMEM106B deficient mice strongly supports a crucial role of TMEM106B in the cerebellum through the regulation of lysosomal functions (Fig. 9): (1) TMEM106B is expressed and localized in the lysosomal compartment in different types of neurons (Additional file 1: Fig. 1); (2) TMEM106B deficiency leads to lysosomal enlargement in DCN neurons (Fig. 5e, f) and the loss of synaptic connections between DCN neurons and Purkinje cells (Fig. 3g, h) at 2 months of age; (3) TMEM106B ablation results in decreased levels of lysosomal protease Cath D in PAVLB-positive interneurons and granule neurons at 2 months of age (Fig. 5a–d); (4) loss of TMEM106B also leads to myelination defects and axon swelling at 2 and 5 months of age, respectively (Fig. 3a–e); (5) ablation of TMEM106B leads to the accumulation of lysosomal vacuoles at the distal end of AIS and death of Purkinje cells during aging (Fig. 4c, d); (6) lysosomes are enlarged and clustered in the microglia of aged TMEM106B knockout mice (Fig. 7a, b). Importantly, a TMEM106B risk allele (rs1990622T) is associated with increased Purkinje cell degeneration in humans (Fig. 8a, b). All these data support that TMEM106B is required for proper lysosomal functions in both neurons and glia and is critical for axon maintenance and synaptic integrity in the cerebellum.

### Lysosomal functions of TMEM106B

TMEM106B is critical for proper lysosomal function [4, 6, 23] and has been shown to regulate several aspects of lysosomal activities, including lysosomal morphology and function [4, 6, 23, 39], lysosome pH [6, 20, 22], lysosome exocytosis [22], lysosomal positioning within the cell [14], lysosomal trafficking in neuronal dendrites [36] and lysosomal trafficking across the axon initial segment (AIS) of axons [13, 25].

Previously we have shown that TMEM106B physically interacts with the lysosomal protease cathepsin D and is required to maintain the protein levels of LAMP1, cathepsin D, and L in oligodendrocytes [14]. In this study, we found that the levels of cathepsin D are significantly decreased in the PVALB-positive interneurons and cerebellar granule neurons, but not in the DCN projection neurons in the cerebellum of 2-month-old *Tmem106b*^*−/−*^ mice (Fig. 5a–d). In addition, lysosomal enlargement has been observed in DCN neurons (Fig. 5e, f) but not in Purkinje cells (data not shown), PVALB-positive interneurons, or granule neurons (Fig. 5a–d). Distinct effects of TMEM106B on lysosomes have also been observed in different types of neurons in the cortex (Fig. 6). Deletion of TMEM106B also induces different lysosomal phenotypes in glia cells in aged mice, with enlarged and clustered lysosomes observed in microglia but not in astrocytes (Fig. 7). Altogether, this suggests that TMEM106B deficiency results in distinct lysosomal phenotypes among different cell types in the brain. Possible explanations for this observation include the cell-type-specific function of lysosomes and potential unique binding partners of TMEM106B expressed in any given cell type. For example, TMEM106B has been reported to regulate lysosomal positioning and trafficking in neuronal dendrites [36] and lysosomal trafficking across the AIS segment of motor neurons [13, 25]. TMEM106B may play a more important role in lysosomal movement and fusion/fission in the soma of DCN neurons and excitatory neurons in the cortex but a more critical role in lysosomal trafficking along axons in Purkinje cells and motor neurons.

### TMEM106B is critical for axonal maintenance and synaptic integrity

Our results have indicated a critical role of TMEM106B in maintaining the proper axonal architecture and synaptic integrity. A significant reduction in SYN signals between Purkinje axons and DCN neurons was detected as early as 2 months in age (Fig. 3g, h). Myelination defects and axonal swelling have been observed in the Purkinje cells of 5-month-old *Tmem106b*^*−/−*^ mice (Fig. 3a–e). Since lysosomal enlargement in the DCN neurons can be detected at 2-month of age (Fig. 5e, f), it is possible that lysosomal defects of DCN neurons together with myelination defects, which can be detected as early as 2 months of age (Fig. 3), trigger the changes in the synaptic connections between Purkinje cells and DCN neurons.

TMEM106B has been shown to affect lysosomal trafficking in neuronal dendrites [36]. Down-regulation of TMEM106B significantly increases retrograde transport of lysosomes in dendrites and reduced dendritic branching [36]. Alterations in dendritic spine density and morphology upon TMEM106B loss could possibly result in changes in synaptic function. In our study, we have not observed any obvious change in dendritic morphlogy in TMEM106B deficient mice, but more detailed analysis is required to reveal subtle perturbations.

### TMEM106B is essential for maintaining the proper function of Purkinje cells

The most striking phenotype of *Tmem106b*^*−/−*^ mice is the degeneration of Purkinje cells in the anterior lobe of the cerebellum during aging. Our data support a model in which TMEM106B modulates lysosomal functions and promotes axonal maintenance and health in Purkinje cells to affect their survival: (1) TMEM106B is highly expressed in Purkinje cells (Additional file 1: Fig. 1); (2) TMEM106B promotes axonal maintenance and health in Purkinje cells, and TMEM106B deficiency in young mice leads to Purkinje axon swelling (Fig. 3c, e) and a reduction of presynaptic marker synaptophysin (SYN), resulting in the loss of synapse between Purkinje axons and DCN neurons in the cerebellum at an early stage (Fig. 3g, h); (3) TMEM106B deficiency leads to de-myelination of Purkinje axons in the young mice (Fig. 3c, h); (4) deletion of TMEM106B leads to enlarged Cath D-positive lysosomal vacuoles at the distal end of the AIS in Purkinje axons in aged mice (Fig. 4c, d); (5) finally, lysosomal abnormalities in microglia are likely to cause reduced clearance of cell debris and abnormal inflammation and synaptic pruning (Fig. 7a, b).

Interestingly, the loss of Purkinje cells in the anterior lobe is accompanied by an increased intensity of Parvalbumin (PAVLB)-positive interneurons (Fig. 1a–d). PAVLB is a calcium-binding protein and is expressed in both interneurons and Purkinje cells [35]. Since PVALB-positive interneurons are derived from progenitors that proliferate in the prospective white matter (PWM) during late embryonic life and postnatal development [9, 51], we further examined whether the number of PVALB-positive interneurons was altered in deep cerebellar nuclei (DCN). Interestingly, PVALB-positive interneurons were also observed in the small region of DCN facing the anterior lobe in TMEM106B-deficient mice (Additional file 1: Fig. 8a, b), and these interneurons are positive for PAX2, a marker for cerebellar inhibitory interneuron progenitors (Additional file 1: Fig. 8c). This indicates that the increase in PVALB-positive interneurons observed in the ML might be due to the differentiation of progenitor cells in the DCN and subsequent migration to ML. The mechanism driving the differentiation of PVALB-positive interneurons in response to Purkinje cell death remains to be determined. It should be noted that an increased density of PAVLB -positive interneurons in the molecular layer has also been reported in mice deficient in GluD2, a member of the δ subfamily of ionotropic glutamate receptors [21]. GluD2 is expressed exclusively at parallel fiber (PF) synapses on cerebellar Purkinje cells (PC) and plays a key role in the formation and maintenance of PF–PC synapses [21]. GluD2 is also weakly expressed in PF synapses on interneuron dendrites [21]. Interestingly, we found that GluD2 protein levels are significantly decreased in the 6- and 16-month-old *Tmem106b*^*−/−*^ mice (Additional file 1: Fig. 9). While we cannot rule out this decrease is the consequence of the loss of Purkinje cells at 16 months of age, it is possible that changes in GluD2 protein levels and activities at 6 months of age affects the formation of PF-PC synapses, which further contribute to the alterations in interneuron density and synapse integrity in *Tmem106b*^*−/−*^ mice.

### The role of the cerebellum in neurodegenerative diseases

TMEM106B has been associated with brain aging and many brain disorders [12]. Interestingly the cell type affected most in *Tmem106b*^*−/−*^ mice is the Purkinje cell. Moreover, the TMEM106B risk allele is associated with an increased level of Purkinje degeneration in humans (Fig. 8). These results strongly support that TMEM106B is closely linked to cerebellar functions. Accumulating evidence supports that the cerebellum plays critical roles in cognition, executive functions [1], emotion processing [18], reward memory, and addiction [28], in addition to its traditionally recognized function in motor coordination and control. In addition, cerebellar dysfunction has been recently tightly associated with FTLD and other neurodegenerative diseases [2, 3, 7, 8, 43]. For example, in FTLD-*C9orf72* patients, the earliest volume changes have been observed in the cerebellum and a few other subcortical regions [3]. Cerebellar atrophy has also been associated with cognition in corticobasal syndrome and progressive supranuclear palsy [43]. Cerebellar dysfunction might also promote the progression of Alzheimer’s disease after general anesthesia [38]. Thus, the effect of TMEM106B in the cerebellum might influence cognitive, executive, and emotional functions during brain aging and the progression of TMEM106B-associated brain disorders.

## Supplementary Information

**Additional file 1. Supplementary Figures 1–9 and Supplementary Table 1**

## Data Availability

The data supporting the findings of this study are available from the corresponding author upon request.
