# Supplementary material for: TMEM106B deficiency impairs cerebellar myelination and synaptic integrity with Purkinje cell loss
Source: Acta Neuropathol Commun. 2022 Mar 14;10:33. doi: 10.1186/s40478-022-01334-7 (PMC8919601; doi:10.1186/s40478-022-01334-7)
Supplement: Supplementary file 1 — Additional file 1. Supplementary Figures 1–9 and Supplementary Table 1 [file 40478_2022_1334_MOESM1_ESM.docx]

**
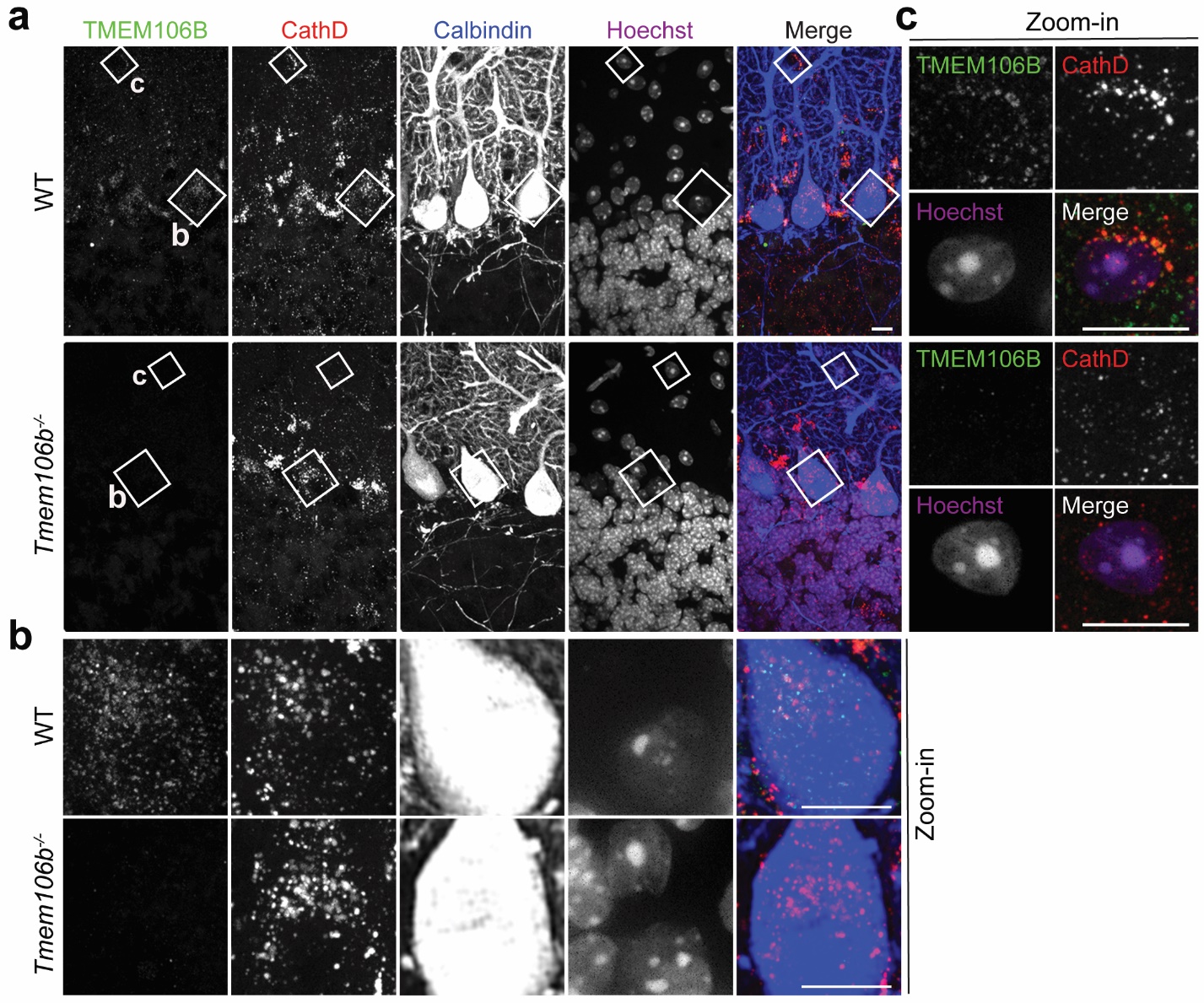
**

**Additional file 1: Fig. 1 Lysosomal localization of TMEM106B in different types of neurons in the cerebellum. (a)** Immunostaining of TMEM106B, Cathepsin D (Cath D), and Calbindin in cerebellar sections from 5-month‐old WT and *Tmem106b^-/-^* mice. Scale bar= 10µm. **(b, c)** Representative images of Purkinje cell (b) and interneurons (c) in the molecular cell layer were shown in zoom-in images. Scale bar= 10 µm.

**
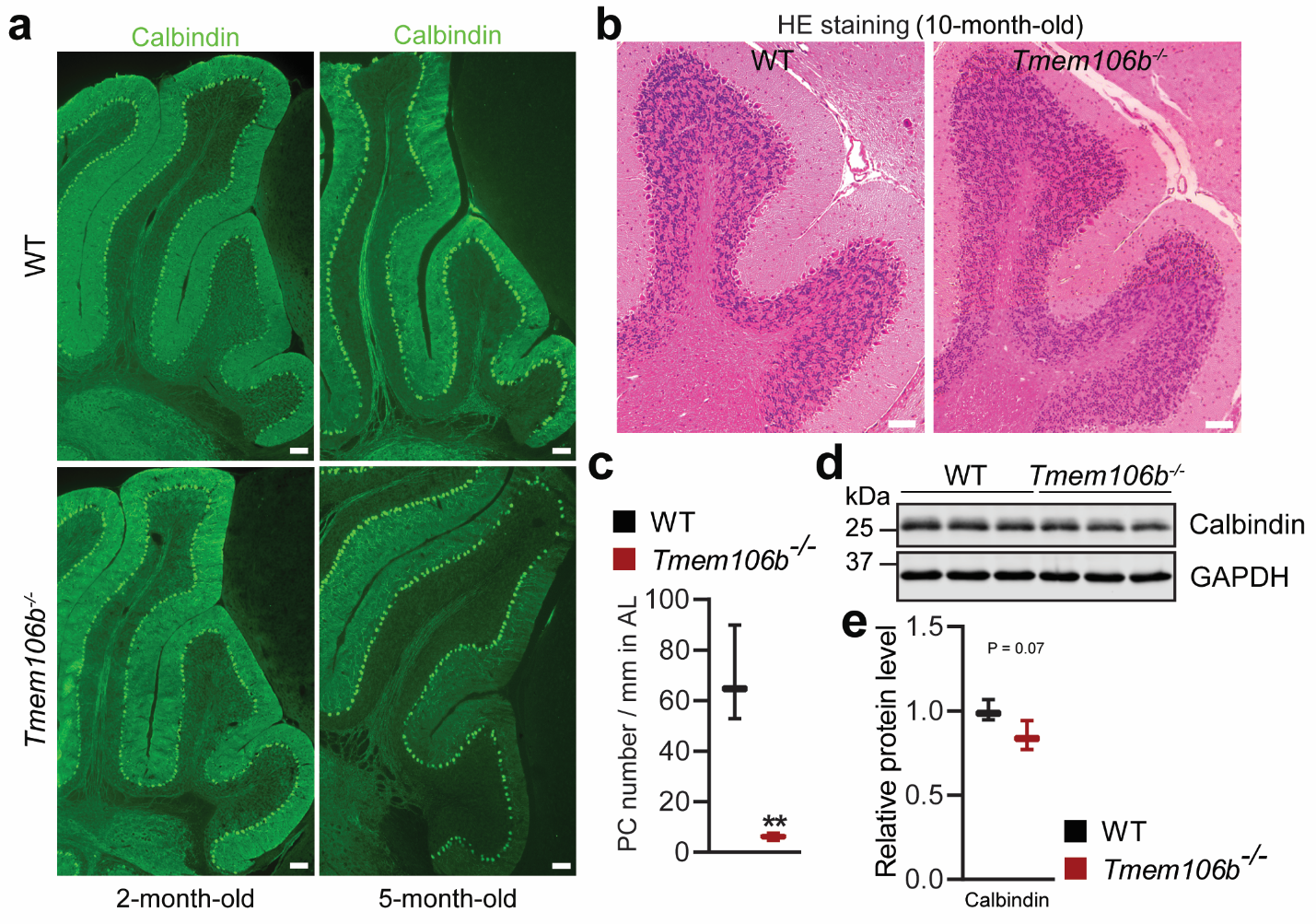
**

**Additional file 1: Fig. 2 TMEM106B deletion does not lead to Purkinje cell degeneration in young mice.**

**(a)** Cerebellar sections from 2- and 5‐month‐old WT and *Tmem106b^-/-^* mice were stained with calbindin antibody. Scale bar = 100 µm. **(b, c)** HE staining of cerebellar sections from 10‐month‐old WT and *Tmem106b^-/-^* mice. Purkinje cell numbers in the anterior lobe (AL) were quantified in (**c**). Scale bar = 100 µm. n=3-4, **, p<0.01, unpaired t-test. **(d, e)** Western blot analysis of Calbindin protein level in 5-month-old WT and *Tmem106b^-/-^* cerebellar lysates. GAPDH was used as an internal control. Relative protein levels were quantified in (**e**). n=3, unpaired t-test.

**
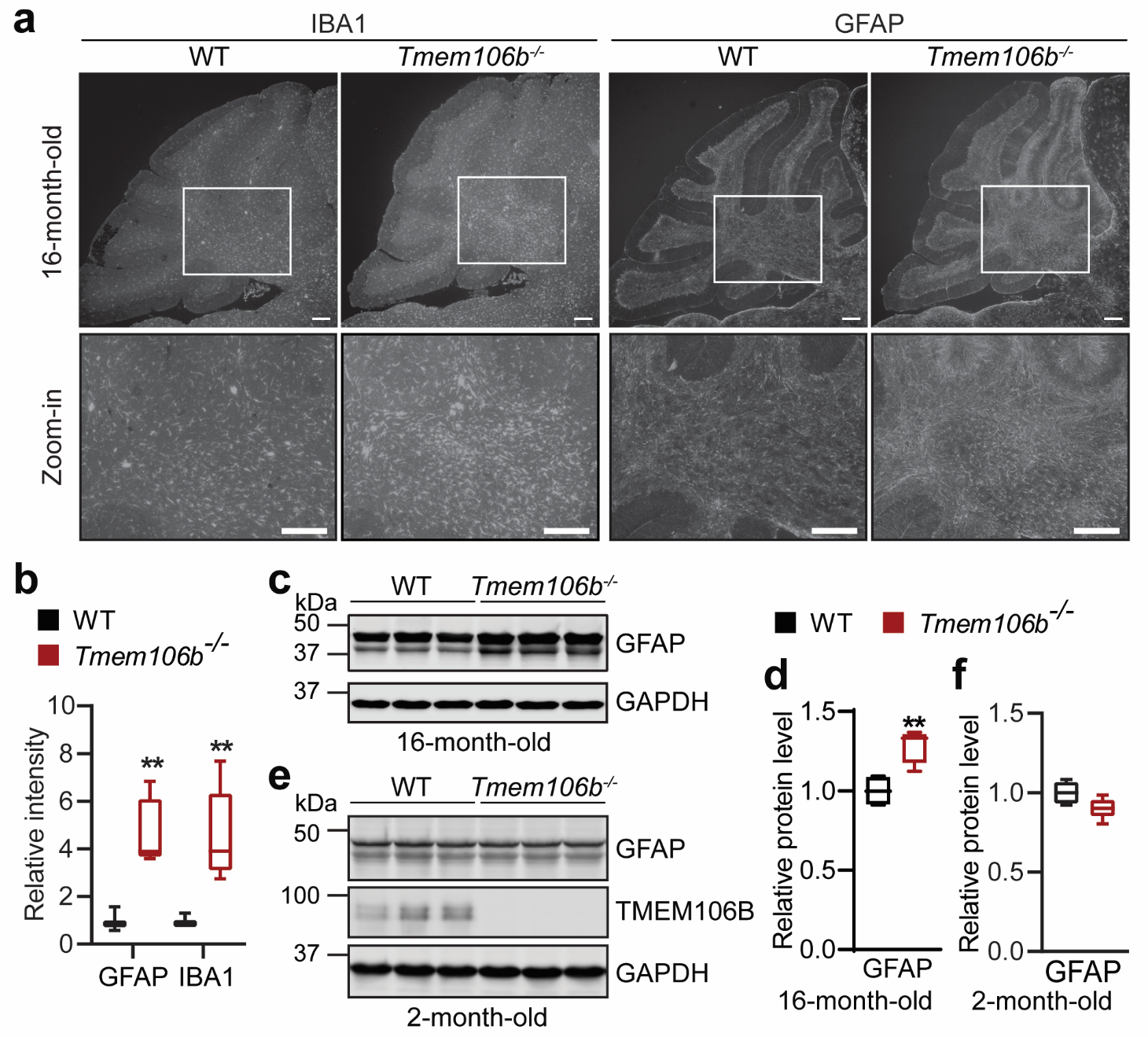
**

**Additional file 1: Fig. 3 Glia activation in the cerebellum of aged *Tmem106b^-/-^* mice**

**(a, b)** Cerebellar sections from 16‐month‐old *Tmem106b^-/-^* mice were stained with anti-IBA1 or GFAP antibodies. Quantification of the intensity of GFAP or IBA1 in (**b**). n=3-4, **, p<0.01, unpaired t-test. Scale bar = 100 µm. **(c-f)** Western blot analysis of GFAP protein levels in WT and *Tmem106b^-/-^* cerebellar lysates (**c,d**: 6-month-old mice; **e,f**: 2-month-old mice). n=5, **, p<0.01, unpaired t-test.

**
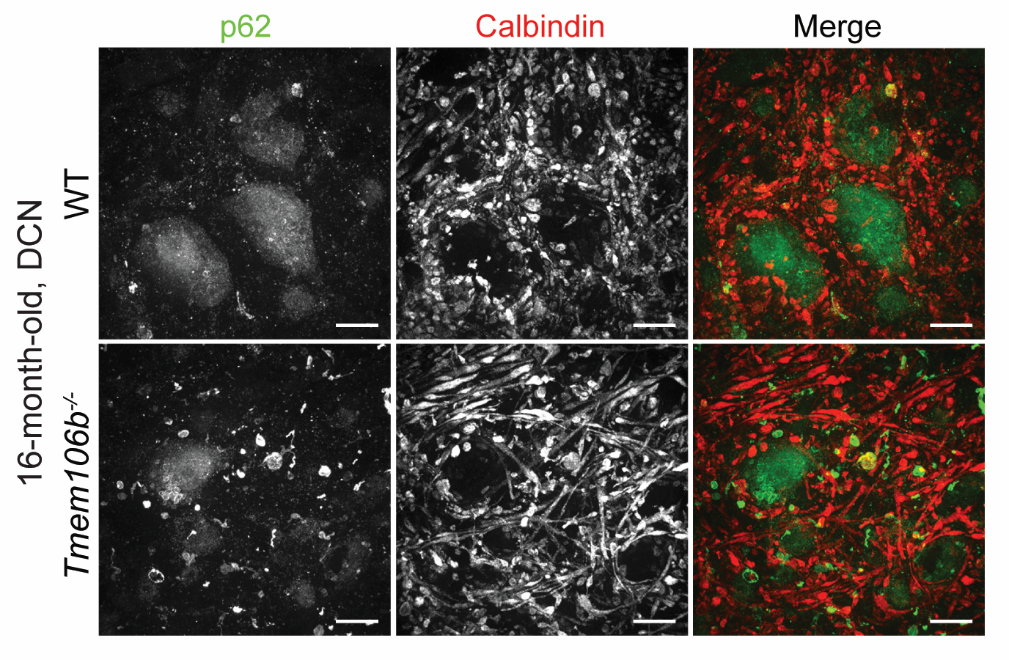
**

**Additional file 1: Fig. 4 p62 positive puncta overlap with calbindin-positive axons of Purkinje cells in the DCN in 16‐month‐old *Tmem106b^-/-^* mice**.

Cerebellar sections from 16‐month‐old *Tmem106b^-/-^* mice were immuno-stained with p62 and Calbindin antibodies, and representative images from the DCN were shown. Scale bar = 100 µm.

**
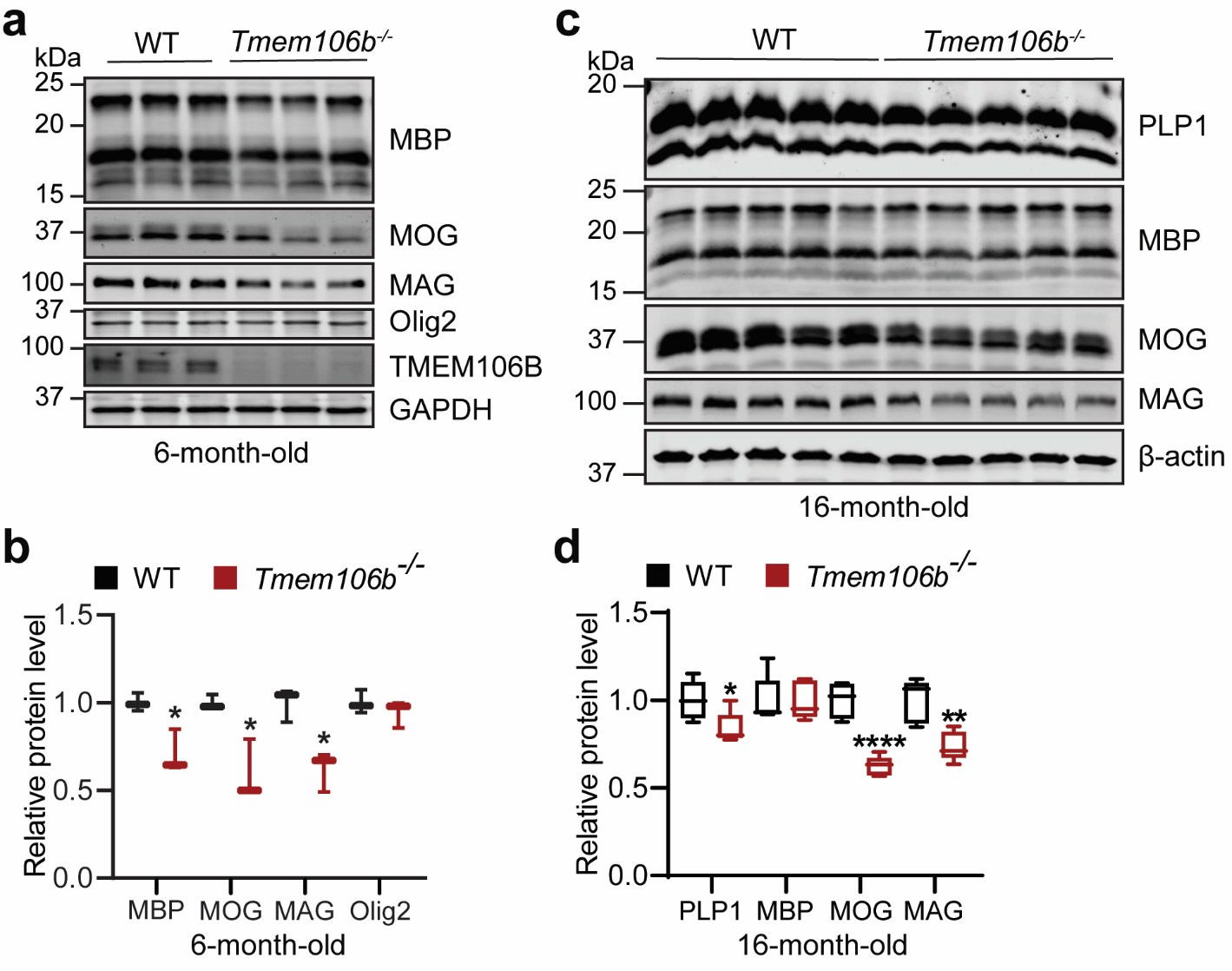
**

**Additional file 1: Fig. 5 Myelination defects in the cerebellum of 5- and 16-month-old *Tmem106b^-/-^* mice**

**(a-d)** Western blot analysis of myelin proteins and GAPDH in 6- and 16-month-old WT and *Tmem106b^-/-^* cerebellar lysates. Protein levels were quantified and normalized to GAPDH in **b** (6-month-old mice, n = 3) and to actin in **d** (16-month-old mice, n = 5), respectively. *, p<0.05, **, p<0.01, ***, p<0.001, unpaired t-test.

**
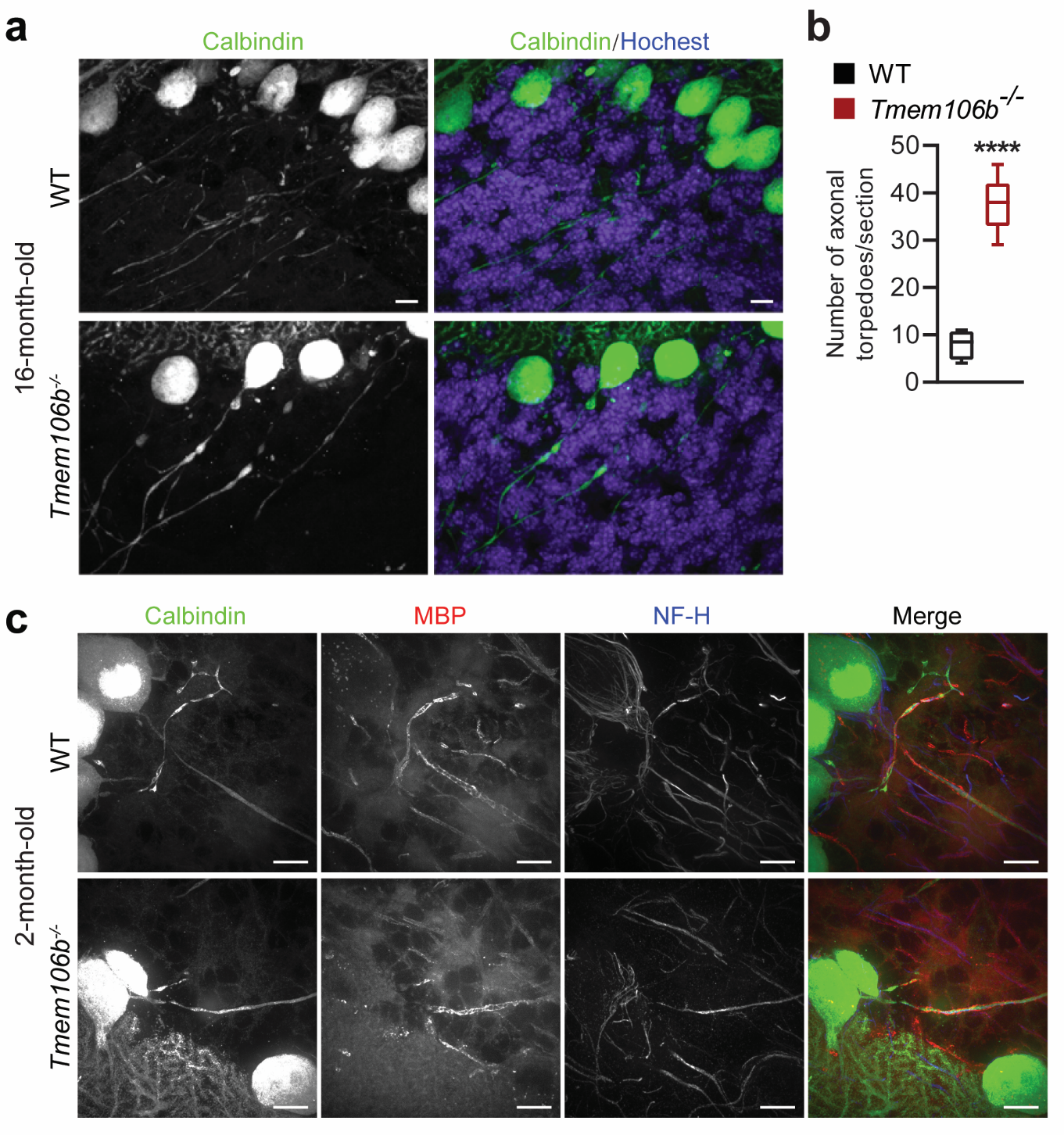
**

**Additional file 1: Fig. 6 Purkinje cell axon swelling in 16-month-old *Tmem106b^-/-^* mice, but not in 2-month-old *Tmem106b^-/-^* mice**

**(a, b)** Cerebellar sections from 16‐month‐old WT and *Tmem106b^-/-^* mice were co-stained with Calbindin antibody and Hoechst. The number of axonal torpedoes per section was quantified in **(b)**. ****, p<0.0001, unpaired t-test. Scale bar = 10 µm. **(c)** Cerebellar sections from 2‐month‐old WT and *Tmem106b^-/-^* mice were co-stained with Calbindin, myelin basic protein (MBP) and NF-H antibodies. Scale bar = 10 µm.

**
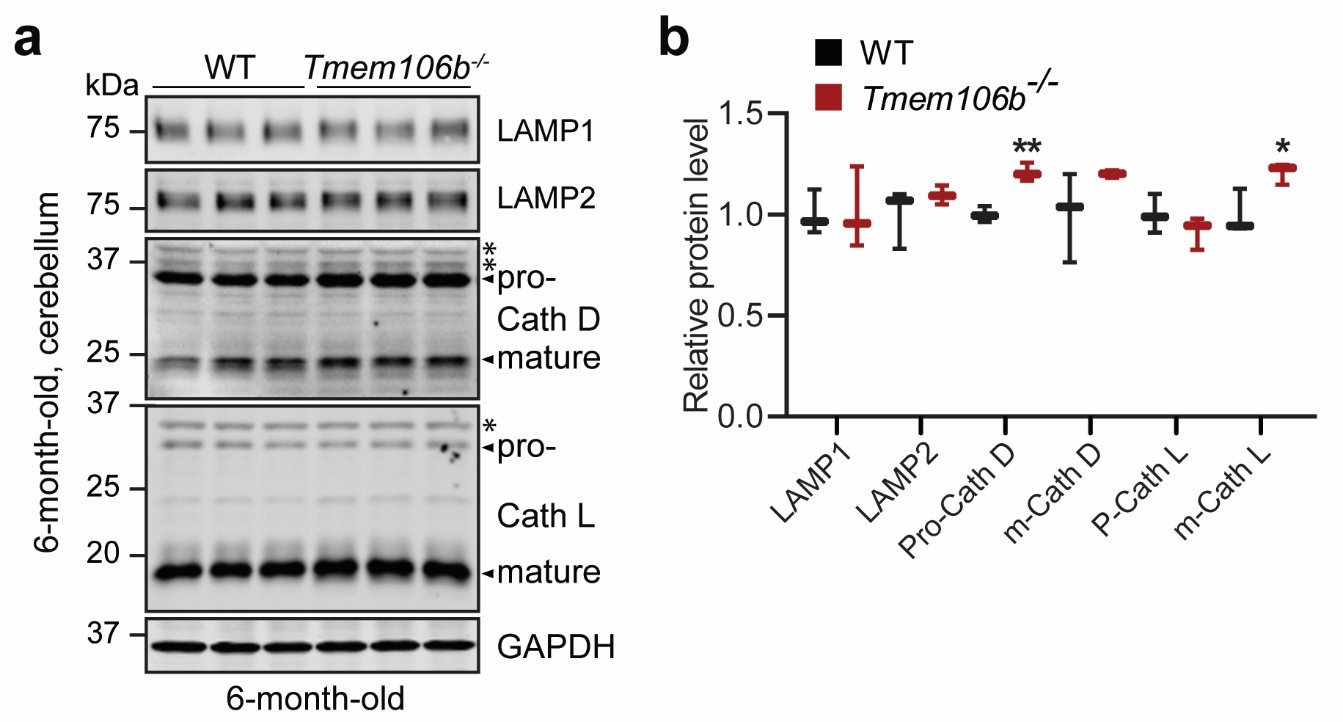
**

**Additional file 1: Fig. 7 Mild upregulation of lysosomal proteins in the cerebellum of 6-month-old *Tmem106b^-/-^* mice**

**(a, b)** Western blot analysis of lysosomal proteins and GAPDH in 6-month-old WT and *Tmem106b^-/-^* cerebellar lysates. Protein levels were quantified and normalized to GAPDH in (**B**). n=3, *, p<0.05, **, p<0.01, unpaired t-test. Asterisk indicates non‐specific bands.

**
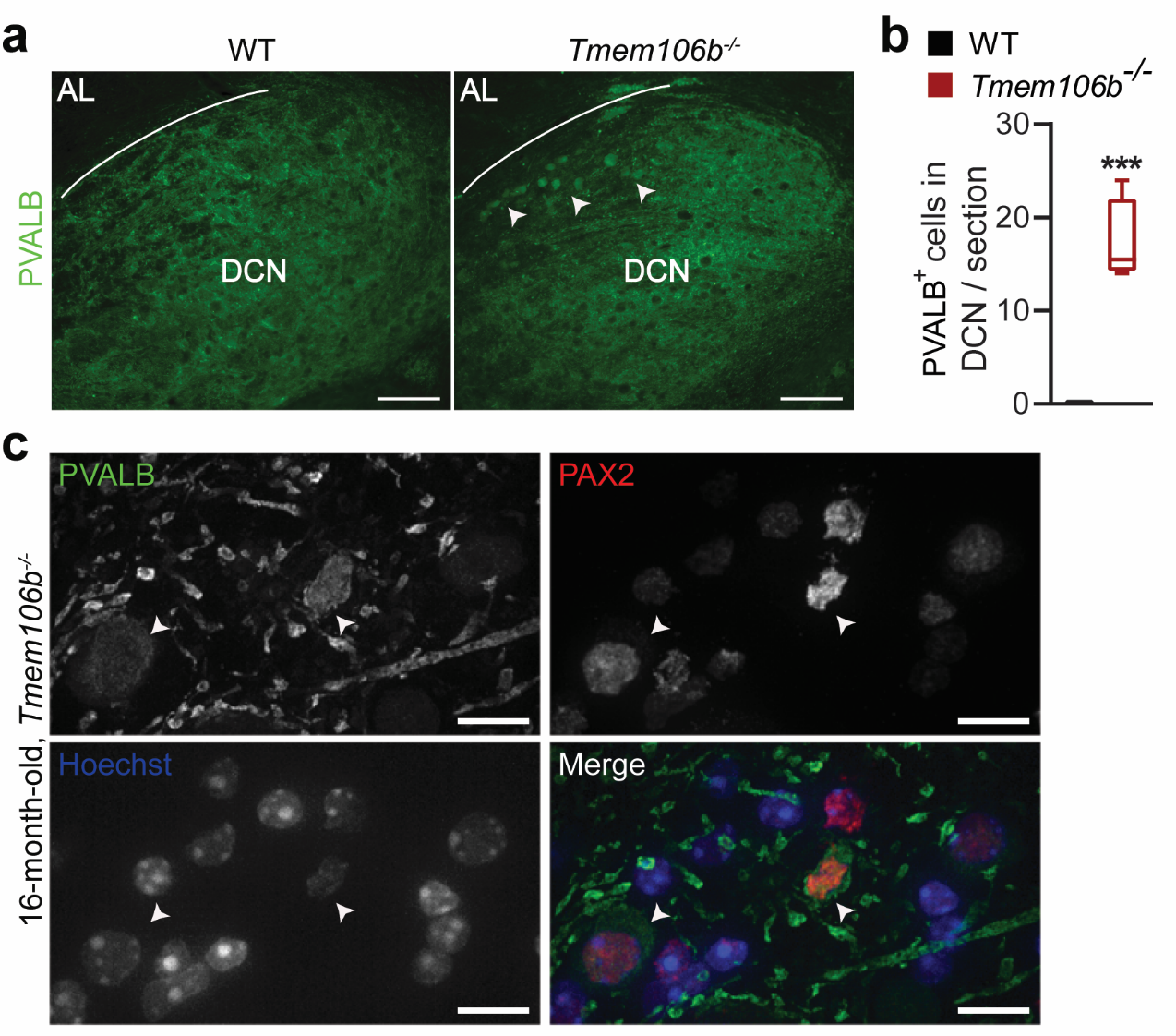
**

**Additional file 1: Fig. 8 PVALB-positive interneurons are present in the small region of DCN facing the anterior lobe in aged TMEM106B-deficient mice**

**(a, b)** Cerebellar sections from 16-month-old WT and *Tmem106b^-/-^* mice were immunostained with anti-parvalbumin (PVALB) antibody. The number of PVALB-positive interneurons in DCN facing the anterior lobe was quantified in (**b**). Scale bar = 100 µm. n=3-4, ***, p<0.001, unpaired t-test. **(c)** Cerebellar sections from 16-month-old *Tmem106b^-/-^* mice were immunostained with parvalbumin (PVALB) and PAX2 antibodies. Nuclei were stained with Hoechst. Scale bar = 10 µm.

**
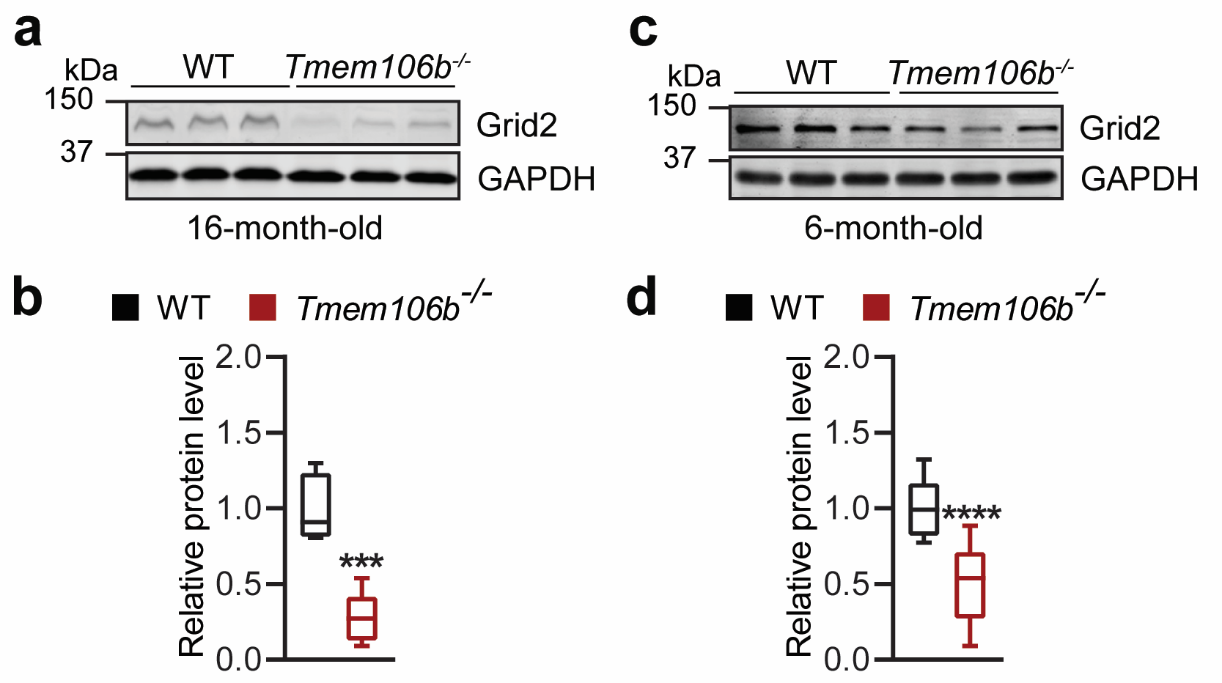
**

**Additional file 1: Fig. 9 Reduction of Grid2 protein level in the cerebellum of 6- and 16-month-old *Tmem106b^-/-^* mice**

**(a-d)** Western blot analysis of Grid2 protein level and GAPDH in 6- (**a,b**) and 16-month-old (**c,d**) WT and *Tmem106b^-/-^* cerebellar lysates. Protein level was quantified and normalized to GAPDH in **b** (6-month-old mice, n = 6) and **d** (16-month-old mice, n = 5), respectively. ***, p<0.001, ****, p<0.0001, unpaired t-test.

**Additional file 1: Table 1: List of human patient samples used in this study.**

| **Sex** | **Age at Death** | **rs1990622** | **Race** | **Ethnicity** | **Tau** | **Diffuse amyloid** | **Clinical Diagnoses** | **Neuropathologic Diagnoses** |
| --- | --- | --- | --- | --- | --- | --- | --- | --- |
| Male | 86 | C/C | White | Not Hispanic or Latino |  | Rare | Parkinson's Disease with Dementia | High ADNC, LBD (limbic), LATE |
| Male | 83 | C/C | White | Not Hispanic or Latino | 0 | 0 | Generalized dystonia | Low ADNC |
| Female | 51 | C/C | Black or African American | Unknown or Not Reported | 0 | 0 | Normal | PART, Metastatic adenocarcinoma |
| Female | 73 | C/C | White | Not Hispanic or Latino | Rare | 1+ | Probable Alzheimer's Disease | High ADNC, LATE |
| Female | 85 | C/C | White | Not Hispanic or Latino | 0 | 1+ | Probable Alzheimer's Disease | High ADNC, LATE |
| Male | 87 | C/C | White | Not Hispanic or Latino | 0 | 0 | Mild cognitive impairment (Amnestic) | Intermediate ADNC |
| Female | 73 | C/C | White | Not Hispanic or Latino | 0 | 0 | FTLD-bvFTD | FTLD-TDP, PART |
| Female | 90+ | C/C | White | Not Hispanic or Latino | 0 | 0 | Normal | Intermediate ADNC, LBD (brainstem) |
| Female | 77 | C/C | White | Not Hispanic or Latino | 0 | 0 | Parkinson's Disease (not demented) | LBD (brainstem) |
| Male | 63 | C/C | White | Not Hispanic or Latino | 0 | 0 | Parkinson's Disease (not demented) | LBD (brainstem), low ADNC |
| Female | 73 | C/C | White | Not Hispanic or Latino | Rare | 1+ | Probable Alzheimer's Disease | High ADNC, LATE |
| Male | 67 | C/T | White | Not Hispanic or Latino |  | 0 | Bipolar disease | PART |
| Female | 88 | C/T | White | Not Hispanic or Latino | 0 | 0 | Probable Alzheimer's Disease | Intermediate ADNC, LBD (brainstem) |
| Male | 68 | C/T | White | Not Hispanic or Latino | 0 | Rare | Probable Alzheimer's Disease | High ADNC |
| Male | 62 | C/T |  |  | 0 | 0 | Dementia with Lewy Bodies | LBD (neocortical), low ADNC |
| Female | 72 | C/T | Black or African American |  | 0 | 0 | Normal | PART, CVD |
| Male | 73 | C/T |  |  | 0 | Rare | Dementia with Lewy Bodies | LBD (limbic), low ADNC |
| Male | 70 | C/T | White | Not Hispanic or Latino | 0 | 0 | Dementia with Lewy Bodies | LBD (neocortical), PART |
| Male | 66 | C/T | White |  | 0 | 0 | Normal | PART |
| Female | 83 | C/T | White | Not Hispanic or Latino | 0 | 0 | Probable Alzheimer's Disease | High ADNC, LATE |
| Male | 72 | C/T | White | Not Hispanic or Latino | 0 | 0 | Dementia with Lewy Bodies | LBD (neocortical), intermediate ADNC |
| Female | 90+ | C/T | White | Not Hispanic or Latino | 0 | 0 | Parkinson's Disease with Dementia | LBD (limbic), intermediate ADNC |
| Male | 70 | C/T | White | Not Hispanic or Latino | 0 | 0 | Parkinson's Disease with Dementia | LBD (neocortical, low ADNC |
| Male | 66 | C/T | White | Not Hispanic or Latino | 0 | 1+ | Dementia with Lewy Bodies | High ADNC |
| Female | 81 | T/T | White | Not Hispanic or Latino | 0 | 0 | Dementia with Lewy Bodies | LBD (neocortical), intermediate ADNC |
| Male | 85 | T/T | White | Not Hispanic or Latino | 0 | Rare | FTLD-NOS | High ADNC, LBD (amygdala) |
| Male | 81 | T/T | White | Not Hispanic or Latino | 0 | 0 | FTLD-bvFTD | PART, LBD (brainstem) |
| Female | 74 | T/T | White | Not Hispanic or Latino | 0 | 1+ | Probable Alzheimer's Disease | High ADNC, LATE, LBD (amygdala) |
| Male | 86 | T/T | White | Not Hispanic or Latino | 0 | Rare | Probable Alzheimer's Disease | High ADNC, LATE, LBD (amygdala) |
| Female | 75 | T/T | White | Not Hispanic or Latino | 0 | Rare | Probable Alzheimer's Disease | High ADNC, LBD (limbic), LATE |
| Male | 83 | T/T | White | Not Hispanic or Latino | 0 | 0 | Probable Alzheimer's Disease | High ADNC, LATE, LBD (amygdala) |
| Male | 86 | T/T | White | Not Hispanic or Latino | 0 | 1+ | Probable Alzheimer's Disease | High ADNC, LATE, HS |
| Male | 65 | T/T | White | Not Hispanic or Latino | 0 | 0 | FTLD-bvFTD | FTLD-TDP, low ADNC |
| Male | 78 | T/T | White | Not Hispanic or Latino | 0 | 0 | FTLD-bvFTD | FTLD-TDP, HS, CVD, Intermediate ADNC |
